# Search for supersymmetry in pp collisions at $\sqrt{s} =7$ TeV in events with a single lepton, jets, and missing transverse momentum

**DOI:** 10.1140/epjc/s10052-013-2404-z

**Published:** 2013-05-08

**Authors:** The CMS Collaboration, S. Chatrchyan, V. Khachatryan, A. M. Sirunyan, A. Tumasyan, W. Adam, E. Aguilo, T. Bergauer, M. Dragicevic, J. Erö, C. Fabjan, M. Friedl, R. Frühwirth, V. M. Ghete, J. Hammer, N. Hörmann, J. Hrubec, M. Jeitler, W. Kiesenhofer, V. Knünz, M. Krammer, I. Krätschmer, D. Liko, I. Mikulec, M. Pernicka, B. Rahbaran, C. Rohringer, H. Rohringer, R. Schöfbeck, J. Strauss, A. Taurok, W. Waltenberger, G. Walzel, E. Widl, C.-E. Wulz, V. Mossolov, N. Shumeiko, J. Suarez Gonzalez, M. Bansal, S. Bansal, T. Cornelis, E. A. De Wolf, X. Janssen, S. Luyckx, L. Mucibello, S. Ochesanu, B. Roland, R. Rougny, M. Selvaggi, Z. Staykova, H. Van Haevermaet, P. Van Mechelen, N. Van Remortel, A. Van Spilbeeck, F. Blekman, S. Blyweert, J. D’Hondt, R. Gonzalez Suarez, A. Kalogeropoulos, M. Maes, A. Olbrechts, W. Van Doninck, P. Van Mulders, G. P. Van Onsem, I. Villella, B. Clerbaux, G. De Lentdecker, V. Dero, A. P. R. Gay, T. Hreus, A. Léonard, P. E. Marage, A. Mohammadi, T. Reis, L. Thomas, G. Vander Marcken, C. Vander Velde, P. Vanlaer, J. Wang, V. Adler, K. Beernaert, A. Cimmino, S. Costantini, G. Garcia, M. Grunewald, B. Klein, J. Lellouch, A. Marinov, J. Mccartin, A. A. Ocampo Rios, D. Ryckbosch, N. Strobbe, F. Thyssen, M. Tytgat, P. Verwilligen, S. Walsh, E. Yazgan, N. Zaganidis, S. Basegmez, G. Bruno, R. Castello, L. Ceard, C. Delaere, T. du Pree, D. Favart, L. Forthomme, A. Giammanco, J. Hollar, V. Lemaitre, J. Liao, O. Militaru, C. Nuttens, D. Pagano, A. Pin, K. Piotrzkowski, N. Schul, J. M. Vizan Garcia, N. Beliy, T. Caebergs, E. Daubie, G. H. Hammad, G. A. Alves, M. Correa Martins Junior, T. Martins, M. E. Pol, M. H. G. Souza, W. L. Aldá Júnior, W. Carvalho, A. Custódio, E. M. Da Costa, D. De Jesus Damiao, C. De Oliveira Martins, S. Fonseca De Souza, D. Matos Figueiredo, L. Mundim, H. Nogima, W. L. Prado Da Silva, A. Santoro, L. Soares Jorge, A. Sznajder, T. S. Anjos, C. A. Bernardes, F. A. Dias, T. R. Fernandez Perez Tomei, E. M. Gregores, C. Lagana, F. Marinho, P. G. Mercadante, S. F. Novaes, Sandra S. Padula, V. Genchev, P. Iaydjiev, S. Piperov, M. Rodozov, S. Stoykova, G. Sultanov, V. Tcholakov, R. Trayanov, M. Vutova, A. Dimitrov, R. Hadjiiska, V. Kozhuharov, L. Litov, B. Pavlov, P. Petkov, J. G. Bian, G. M. Chen, H. S. Chen, C. H. Jiang, D. Liang, S. Liang, X. Meng, J. Tao, J. Wang, X. Wang, Z. Wang, H. Xiao, M. Xu, J. Zang, Z. Zhang, C. Asawatangtrakuldee, Y. Ban, Y. Guo, W. Li, S. Liu, Y. Mao, S. J. Qian, H. Teng, D. Wang, L. Zhang, W. Zou, C. Avila, J. P. Gomez, B. Gomez Moreno, A. F. Osorio Oliveros, J. C. Sanabria, N. Godinovic, D. Lelas, R. Plestina, D. Polic, I. Puljak, Z. Antunovic, M. Kovac, V. Brigljevic, S. Duric, K. Kadija, J. Luetic, S. Morovic, A. Attikis, M. Galanti, G. Mavromanolakis, J. Mousa, C. Nicolaou, F. Ptochos, P. A. Razis, M. Finger, M. Finger, Y. Assran, S. Elgammal, A. Ellithi Kamel, M. A. Mahmoud, A. Radi, M. Kadastik, M. Müntel, M. Raidal, L. Rebane, A. Tiko, P. Eerola, G. Fedi, M. Voutilainen, J. Härkönen, A. Heikkinen, V. Karimäki, R. Kinnunen, M. J. Kortelainen, T. Lampén, K. Lassila-Perini, S. Lehti, T. Lindén, P. Luukka, T. Mäenpää, T. Peltola, E. Tuominen, J. Tuominiemi, E. Tuovinen, D. Ungaro, L. Wendland, K. Banzuzi, A. Karjalainen, A. Korpela, T. Tuuva, M. Besancon, S. Choudhury, M. Dejardin, D. Denegri, B. Fabbro, J. L. Faure, F. Ferri, S. Ganjour, A. Givernaud, P. Gras, G. Hamel de Monchenault, P. Jarry, E. Locci, J. Malcles, L. Millischer, A. Nayak, J. Rander, A. Rosowsky, I. Shreyber, M. Titov, S. Baffioni, F. Beaudette, L. Benhabib, L. Bianchini, M. Bluj, C. Broutin, P. Busson, C. Charlot, N. Daci, T. Dahms, M. Dalchenko, L. Dobrzynski, R. Granier de Cassagnac, M. Haguenauer, P. Miné, C. Mironov, I. N. Naranjo, M. Nguyen, C. Ochando, P. Paganini, D. Sabes, R. Salerno, Y. Sirois, C. Veelken, A. Zabi, J.-L. Agram, J. Andrea, D. Bloch, D. Bodin, J.-M. Brom, M. Cardaci, E. C. Chabert, C. Collard, E. Conte, F. Drouhin, C. Ferro, J.-C. Fontaine, D. Gelé, U. Goerlach, P. Juillot, A.-C. Le Bihan, P. Van Hove, F. Fassi, D. Mercier, S. Beauceron, N. Beaupere, O. Bondu, G. Boudoul, J. Chasserat, R. Chierici, D. Contardo, P. Depasse, H. El Mamouni, J. Fay, S. Gascon, M. Gouzevitch, B. Ille, T. Kurca, M. Lethuillier, L. Mirabito, S. Perries, L. Sgandurra, V. Sordini, Y. Tschudi, P. Verdier, S. Viret, Z. Tsamalaidze, G. Anagnostou, C. Autermann, S. Beranek, M. Edelhoff, L. Feld, N. Heracleous, O. Hindrichs, R. Jussen, K. Klein, J. Merz, A. Ostapchuk, A. Perieanu, F. Raupach, J. Sammet, S. Schael, D. Sprenger, H. Weber, B. Wittmer, V. Zhukov, M. Ata, J. Caudron, E. Dietz-Laursonn, D. Duchardt, M. Erdmann, R. Fischer, A. Güth, T. Hebbeker, C. Heidemann, K. Hoepfner, D. Klingebiel, P. Kreuzer, M. Merschmeyer, A. Meyer, M. Olschewski, P. Papacz, H. Pieta, H. Reithler, S. A. Schmitz, L. Sonnenschein, J. Steggemann, D. Teyssier, M. Weber, M. Bontenackels, V. Cherepanov, Y. Erdogan, G. Flügge, H. Geenen, M. Geisler, W. Haj Ahmad, F. Hoehle, B. Kargoll, T. Kress, Y. Kuessel, J. Lingemann, A. Nowack, L. Perchalla, O. Pooth, P. Sauerland, A. Stahl, M. Aldaya Martin, J. Behr, W. Behrenhoff, U. Behrens, M. Bergholz, A. Bethani, K. Borras, A. Burgmeier, A. Cakir, L. Calligaris, A. Campbell, E. Castro, F. Costanza, D. Dammann, C. Diez Pardos, G. Eckerlin, D. Eckstein, G. Flucke, A. Geiser, I. Glushkov, P. Gunnellini, S. Habib, J. Hauk, G. Hellwig, H. Jung, M. Kasemann, P. Katsas, C. Kleinwort, H. Kluge, A. Knutsson, M. Krämer, D. Krücker, E. Kuznetsova, W. Lange, W. Lohmann, B. Lutz, R. Mankel, I. Marfin, M. Marienfeld, I.-A. Melzer-Pellmann, A. B. Meyer, J. Mnich, A. Mussgiller, S. Naumann-Emme, O. Novgorodova, J. Olzem, H. Perrey, A. Petrukhin, D. Pitzl, A. Raspereza, P. M. Ribeiro Cipriano, C. Riedl, E. Ron, M. Rosin, J. Salfeld-Nebgen, R. Schmidt, T. Schoerner-Sadenius, N. Sen, A. Spiridonov, M. Stein, R. Walsh, C. Wissing, V. Blobel, J. Draeger, H. Enderle, J. Erfle, U. Gebbert, M. Görner, T. Hermanns, R. S. Höing, K. Kaschube, G. Kaussen, H. Kirschenmann, R. Klanner, J. Lange, B. Mura, F. Nowak, T. Peiffer, N. Pietsch, D. Rathjens, C. Sander, H. Schettler, P. Schleper, E. Schlieckau, A. Schmidt, M. Schröder, T. Schum, M. Seidel, J. Sibille, V. Sola, H. Stadie, G. Steinbrück, J. Thomsen, L. Vanelderen, C. Barth, J. Berger, C. Böser, T. Chwalek, W. De Boer, A. Descroix, A. Dierlamm, M. Feindt, M. Guthoff, C. Hackstein, F. Hartmann, T. Hauth, M. Heinrich, H. Held, K. H. Hoffmann, U. Husemann, I. Katkov, J. R. Komaragiri, P. Lobelle Pardo, D. Martschei, S. Mueller, Th. Müller, M. Niegel, A. Nürnberg, O. Oberst, A. Oehler, J. Ott, G. Quast, K. Rabbertz, F. Ratnikov, N. Ratnikova, S. Röcker, F.-P. Schilling, G. Schott, H. J. Simonis, F. M. Stober, D. Troendle, R. Ulrich, J. Wagner-Kuhr, S. Wayand, T. Weiler, M. Zeise, G. Daskalakis, T. Geralis, S. Kesisoglou, A. Kyriakis, D. Loukas, I. Manolakos, A. Markou, C. Markou, C. Mavrommatis, E. Ntomari, L. Gouskos, T. J. Mertzimekis, A. Panagiotou, N. Saoulidou, I. Evangelou, C. Foudas, P. Kokkas, N. Manthos, I. Papadopoulos, V. Patras, G. Bencze, C. Hajdu, P. Hidas, D. Horvath, F. Sikler, V. Veszpremi, G. Vesztergombi, N. Beni, S. Czellar, J. Molnar, J. Palinkas, Z. Szillasi, J. Karancsi, P. Raics, Z. L. Trocsanyi, B. Ujvari, S. B. Beri, V. Bhatnagar, N. Dhingra, R. Gupta, M. Kaur, M. Z. Mehta, N. Nishu, L. K. Saini, A. Sharma, J. B. Singh, Ashok Kumar, Arun Kumar, S. Ahuja, A. Bhardwaj, B. C. Choudhary, S. Malhotra, M. Naimuddin, K. Ranjan, V. Sharma, R. K. Shivpuri, S. Banerjee, S. Bhattacharya, S. Dutta, B. Gomber, Sa. Jain, Sh. Jain, R. Khurana, S. Sarkar, M. Sharan, A. Abdulsalam, R. K. Choudhury, D. Dutta, S. Kailas, V. Kumar, P. Mehta, A. K. Mohanty, L. M. Pant, P. Shukla, T. Aziz, S. Ganguly, M. Guchait, M. Maity, G. Majumder, K. Mazumdar, G. B. Mohanty, B. Parida, K. Sudhakar, N. Wickramage, S. Banerjee, S. Dugad, H. Arfaei, H. Bakhshiansohi, S. M. Etesami, A. Fahim, M. Hashemi, H. Hesari, A. Jafari, M. Khakzad, M. Mohammadi Najafabadi, S. Paktinat Mehdiabadi, B. Safarzadeh, M. Zeinali, M. Abbrescia, L. Barbone, C. Calabria, S. S. Chhibra, A. Colaleo, D. Creanza, N. De Filippis, M. De Palma, L. Fiore, G. Iaselli, G. Maggi, M. Maggi, B. Marangelli, S. My, S. Nuzzo, N. Pacifico, A. Pompili, G. Pugliese, G. Selvaggi, L. Silvestris, G. Singh, R. Venditti, G. Zito, G. Abbiendi, A. C. Benvenuti, D. Bonacorsi, S. Braibant-Giacomelli, L. Brigliadori, P. Capiluppi, A. Castro, F. R. Cavallo, M. Cuffiani, G. M. Dallavalle, F. Fabbri, A. Fanfani, D. Fasanella, P. Giacomelli, C. Grandi, L. Guiducci, S. Marcellini, G. Masetti, M. Meneghelli, A. Montanari, F. L. Navarria, F. Odorici, A. Perrotta, F. Primavera, A. M. Rossi, T. Rovelli, G. P. Siroli, R. Travaglini, S. Albergo, G. Cappello, M. Chiorboli, S. Costa, R. Potenza, A. Tricomi, C. Tuve, G. Barbagli, V. Ciulli, C. Civinini, R. D’Alessandro, E. Focardi, S. Frosali, E. Gallo, S. Gonzi, M. Meschini, S. Paoletti, G. Sguazzoni, A. Tropiano, L. Benussi, S. Bianco, S. Colafranceschi, F. Fabbri, D. Piccolo, P. Fabbricatore, R. Musenich, S. Tosi, A. Benaglia, F. De Guio, L. Di Matteo, S. Fiorendi, S. Gennai, A. Ghezzi, S. Malvezzi, R. A. Manzoni, A. Martelli, A. Massironi, D. Menasce, L. Moroni, M. Paganoni, D. Pedrini, S. Ragazzi, N. Redaelli, S. Sala, T. Tabarelli de Fatis, S. Buontempo, C. A. Carrillo Montoya, N. Cavallo, A. De Cosa, O. Dogangun, F. Fabozzi, A. O. M. Iorio, L. Lista, S. Meola, M. Merola, P. Paolucci, P. Azzi, N. Bacchetta, D. Bisello, A. Branca, R. Carlin, P. Checchia, T. Dorigo, U. Dosselli, F. Gasparini, U. Gasparini, A. Gozzelino, K. Kanishchev, S. Lacaprara, I. Lazzizzera, M. Margoni, A. T. Meneguzzo, J. Pazzini, N. Pozzobon, P. Ronchese, F. Simonetto, E. Torassa, M. Tosi, S. Vanini, P. Zotto, G. Zumerle, M. Gabusi, S. P. Ratti, C. Riccardi, P. Torre, P. Vitulo, M. Biasini, G. M. Bilei, L. Fanò, P. Lariccia, G. Mantovani, M. Menichelli, A. Nappi, F. Romeo, A. Saha, A. Santocchia, A. Spiezia, S. Taroni, P. Azzurri, G. Bagliesi, J. Bernardini, T. Boccali, G. Broccolo, R. Castaldi, R. T. D’Agnolo, R. Dell’Orso, F. Fiori, L. Foà, A. Giassi, A. Kraan, F. Ligabue, T. Lomtadze, L. Martini, A. Messineo, F. Palla, A. Rizzi, A. T. Serban, P. Spagnolo, P. Squillacioti, R. Tenchini, G. Tonelli, A. Venturi, P. G. Verdini, L. Barone, F. Cavallari, D. Del Re, M. Diemoz, C. Fanelli, M. Grassi, E. Longo, P. Meridiani, F. Micheli, S. Nourbakhsh, G. Organtini, R. Paramatti, S. Rahatlou, M. Sigamani, L. Soffi, N. Amapane, R. Arcidiacono, S. Argiro, M. Arneodo, C. Biino, N. Cartiglia, M. Costa, N. Demaria, C. Mariotti, S. Maselli, E. Migliore, V. Monaco, M. Musich, M. M. Obertino, N. Pastrone, M. Pelliccioni, A. Potenza, A. Romero, M. Ruspa, R. Sacchi, A. Solano, A. Staiano, A. Vilela Pereira, S. Belforte, V. Candelise, M. Casarsa, F. Cossutti, G. Della Ricca, B. Gobbo, M. Marone, D. Montanino, A. Penzo, A. Schizzi, S. G. Heo, T. Y. Kim, S. K. Nam, S. Chang, D. H. Kim, G. N. Kim, D. J. Kong, H. Park, S. R. Ro, D. C. Son, T. Son, J. Y. Kim, Zero J. Kim, S. Song, S. Choi, D. Gyun, B. Hong, M. Jo, H. Kim, T. J. Kim, K. S. Lee, D. H. Moon, S. K. Park, M. Choi, J. H. Kim, C. Park, I. C. Park, S. Park, G. Ryu, Y. Cho, Y. Choi, Y. K. Choi, J. Goh, M. S. Kim, E. Kwon, B. Lee, J. Lee, S. Lee, H. Seo, I. Yu, M. J. Bilinskas, I. Grigelionis, M. Janulis, A. Juodagalvis, H. Castilla-Valdez, E. De La Cruz-Burelo, I. Heredia-de La Cruz, R. Lopez-Fernandez, R. Magaña Villalba, J. Martínez-Ortega, A. Sanchez-Hernandez, L. M. Villasenor-Cendejas, S. Carrillo Moreno, F. Vazquez Valencia, H. A. Salazar Ibarguen, E. Casimiro Linares, A. Morelos Pineda, M. A. Reyes-Santos, D. Krofcheck, A. J. Bell, P. H. Butler, R. Doesburg, S. Reucroft, H. Silverwood, M. Ahmad, M. H. Ansari, M. I. Asghar, J. Butt, H. R. Hoorani, S. Khalid, W. A. Khan, T. Khurshid, S. Qazi, M. A. Shah, M. Shoaib, H. Bialkowska, B. Boimska, T. Frueboes, R. Gokieli, M. Górski, M. Kazana, K. Nawrocki, K. Romanowska-Rybinska, M. Szleper, G. Wrochna, P. Zalewski, G. Brona, K. Bunkowski, M. Cwiok, W. Dominik, K. Doroba, A. Kalinowski, M. Konecki, J. Krolikowski, N. Almeida, P. Bargassa, A. David, P. Faccioli, P. G. Ferreira Parracho, M. Gallinaro, J. Seixas, J. Varela, P. Vischia, I. Belotelov, P. Bunin, I. Golutvin, V. Karjavin, V. Konoplyanikov, G. Kozlov, A. Lanev, A. Malakhov, P. Moisenz, V. Palichik, V. Perelygin, M. Savina, S. Shmatov, S. Shulha, V. Smirnov, A. Volodko, A. Zarubin, S. Evstyukhin, V. Golovtsov, Y. Ivanov, V. Kim, P. Levchenko, V. Murzin, V. Oreshkin, I. Smirnov, V. Sulimov, L. Uvarov, S. Vavilov, A. Vorobyev, An. Vorobyev, Yu. Andreev, A. Dermenev, S. Gninenko, N. Golubev, M. Kirsanov, N. Krasnikov, V. Matveev, A. Pashenkov, D. Tlisov, A. Toropin, V. Epshteyn, M. Erofeeva, V. Gavrilov, M. Kossov, N. Lychkovskaya, V. Popov, G. Safronov, S. Semenov, V. Stolin, E. Vlasov, A. Zhokin, A. Belyaev, E. Boos, M. Dubinin, L. Dudko, A. Ershov, A. Gribushin, V. Klyukhin, O. Kodolova, I. Lokhtin, A. Markina, S. Obraztsov, M. Perfilov, S. Petrushanko, A. Popov, L. Sarycheva, V. Savrin, A. Snigirev, V. Andreev, M. Azarkin, I. Dremin, M. Kirakosyan, A. Leonidov, G. Mesyats, S. V. Rusakov, A. Vinogradov, I. Azhgirey, I. Bayshev, S. Bitioukov, V. Grishin, V. Kachanov, D. Konstantinov, V. Krychkine, V. Petrov, R. Ryutin, A. Sobol, L. Tourtchanovitch, S. Troshin, N. Tyurin, A. Uzunian, A. Volkov, P. Adzic, M. Djordjevic, M. Ekmedzic, D. Krpic, J. Milosevic, M. Aguilar-Benitez, J. Alcaraz Maestre, P. Arce, C. Battilana, E. Calvo, M. Cerrada, M. Chamizo Llatas, N. Colino, B. De La Cruz, A. Delgado Peris, D. Domínguez Vázquez, C. Fernandez Bedoya, J. P. Fernández Ramos, A. Ferrando, J. Flix, M. C. Fouz, P. Garcia-Abia, O. Gonzalez Lopez, S. Goy Lopez, J. M. Hernandez, M. I. Josa, G. Merino, J. Puerta Pelayo, A. Quintario Olmeda, I. Redondo, L. Romero, J. Santaolalla, M. S. Soares, C. Willmott, C. Albajar, G. Codispoti, J. F. de Trocóniz, H. Brun, J. Cuevas, J. Fernandez Menendez, S. Folgueras, I. Gonzalez Caballero, L. Lloret Iglesias, J. Piedra Gomez, J. A. Brochero Cifuentes, I. J. Cabrillo, A. Calderon, S. H. Chuang, J. Duarte Campderros, M. Felcini, M. Fernandez, G. Gomez, J. Gonzalez Sanchez, A. Graziano, C. Jorda, A. Lopez Virto, J. Marco, R. Marco, C. Martinez Rivero, F. Matorras, F. J. Munoz Sanchez, T. Rodrigo, A. Y. Rodríguez-Marrero, A. Ruiz-Jimeno, L. Scodellaro, I. Vila, R. Vilar Cortabitarte, D. Abbaneo, E. Auffray, G. Auzinger, M. Bachtis, P. Baillon, A. H. Ball, D. Barney, J. F. Benitez, C. Bernet, G. Bianchi, P. Bloch, A. Bocci, A. Bonato, C. Botta, H. Breuker, T. Camporesi, G. Cerminara, T. Christiansen, J. A. Coarasa Perez, D. D’Enterria, A. Dabrowski, A. De Roeck, S. Di Guida, M. Dobson, N. Dupont-Sagorin, A. Elliott-Peisert, B. Frisch, W. Funk, G. Georgiou, M. Giffels, D. Gigi, K. Gill, D. Giordano, M. Girone, M. Giunta, F. Glege, R. Gomez-Reino Garrido, P. Govoni, S. Gowdy, R. Guida, M. Hansen, P. Harris, C. Hartl, J. Harvey, B. Hegner, A. Hinzmann, V. Innocente, P. Janot, K. Kaadze, E. Karavakis, K. Kousouris, P. Lecoq, Y.-J. Lee, P. Lenzi, C. Lourenço, N. Magini, T. Mäki, M. Malberti, L. Malgeri, M. Mannelli, L. Masetti, F. Meijers, S. Mersi, E. Meschi, R. Moser, M. U. Mozer, M. Mulders, P. Musella, E. Nesvold, T. Orimoto, L. Orsini, E. Palencia Cortezon, E. Perez, L. Perrozzi, A. Petrilli, A. Pfeiffer, M. Pierini, M. Pimiä, D. Piparo, G. Polese, L. Quertenmont, A. Racz, W. Reece, J. Rodrigues Antunes, G. Rolandi, C. Rovelli, M. Rovere, H. Sakulin, F. Santanastasio, C. Schäfer, C. Schwick, I. Segoni, S. Sekmen, A. Sharma, P. Siegrist, P. Silva, M. Simon, P. Sphicas, D. Spiga, A. Tsirou, G. I. Veres, J. R. Vlimant, H. K. Wöhri, S. D. Worm, W. D. Zeuner, W. Bertl, K. Deiters, W. Erdmann, K. Gabathuler, R. Horisberger, Q. Ingram, H. C. Kaestli, S. König, D. Kotlinski, U. Langenegger, F. Meier, D. Renker, T. Rohe, L. Bäni, P. Bortignon, M. A. Buchmann, B. Casal, N. Chanon, A. Deisher, G. Dissertori, M. Dittmar, M. Donegà, M. Dünser, J. Eugster, K. Freudenreich, C. Grab, D. Hits, P. Lecomte, W. Lustermann, A. C. Marini, P. Martinez Ruiz del Arbol, N. Mohr, F. Moortgat, C. Nägeli, P. Nef, F. Nessi-Tedaldi, F. Pandolfi, L. Pape, F. Pauss, M. Peruzzi, F. J. Ronga, M. Rossini, L. Sala, A. K. Sanchez, A. Starodumov, B. Stieger, M. Takahashi, L. Tauscher, A. Thea, K. Theofilatos, D. Treille, C. Urscheler, R. Wallny, H. A. Weber, L. Wehrli, C. Amsler, V. Chiochia, S. De Visscher, C. Favaro, M. Ivova Rikova, B. Millan Mejias, P. Otiougova, P. Robmann, H. Snoek, S. Tupputi, M. Verzetti, Y. H. Chang, K. H. Chen, C. M. Kuo, S. W. Li, W. Lin, Z. K. Liu, Y. J. Lu, D. Mekterovic, A. P. Singh, R. Volpe, S. S. Yu, P. Bartalini, P. Chang, Y. H. Chang, Y. W. Chang, Y. Chao, K. F. Chen, C. Dietz, U. Grundler, W.-S. Hou, Y. Hsiung, K. Y. Kao, Y. J. Lei, R.-S. Lu, D. Majumder, E. Petrakou, X. Shi, J. G. Shiu, Y. M. Tzeng, X. Wan, M. Wang, B. Asavapibhop, N. Srimanobhas, A. Adiguzel, M. N. Bakirci, S. Cerci, C. Dozen, I. Dumanoglu, E. Eskut, S. Girgis, G. Gokbulut, E. Gurpinar, I. Hos, E. E. Kangal, T. Karaman, G. Karapinar, A. Kayis Topaksu, G. Onengut, K. Ozdemir, S. Ozturk, A. Polatoz, K. Sogut, D. Sunar Cerci, B. Tali, H. Topakli, L. N. Vergili, M. Vergili, I. V. Akin, T. Aliev, B. Bilin, S. Bilmis, M. Deniz, H. Gamsizkan, A. M. Guler, K. Ocalan, A. Ozpineci, M. Serin, R. Sever, U. E. Surat, M. Yalvac, E. Yildirim, M. Zeyrek, E. Gülmez, B. Isildak, M. Kaya, O. Kaya, S. Ozkorucuklu, N. Sonmez, K. Cankocak, L. Levchuk, J. J. Brooke, E. Clement, D. Cussans, H. Flacher, R. Frazier, J. Goldstein, M. Grimes, G. P. Heath, H. F. Heath, L. Kreczko, S. Metson, D. M. Newbold, K. Nirunpong, A. Poll, S. Senkin, V. J. Smith, T. Williams, L. Basso, K. W. Bell, A. Belyaev, C. Brew, R. M. Brown, D. J. A. Cockerill, J. A. Coughlan, K. Harder, S. Harper, J. Jackson, B. W. Kennedy, E. Olaiya, D. Petyt, B. C. Radburn-Smith, C. H. Shepherd-Themistocleous, I. R. Tomalin, W. J. Womersley, R. Bainbridge, G. Ball, R. Beuselinck, O. Buchmuller, D. Colling, N. Cripps, M. Cutajar, P. Dauncey, G. Davies, M. Della Negra, W. Ferguson, J. Fulcher, D. Futyan, A. Gilbert, A. Guneratne Bryer, G. Hall, Z. Hatherell, J. Hays, G. Iles, M. Jarvis, G. Karapostoli, L. Lyons, A.-M. Magnan, J. Marrouche, B. Mathias, R. Nandi, J. Nash, A. Nikitenko, A. Papageorgiou, J. Pela, M. Pesaresi, K. Petridis, M. Pioppi, D. M. Raymond, S. Rogerson, A. Rose, M. J. Ryan, C. Seez, P. Sharp, A. Sparrow, M. Stoye, A. Tapper, M. Vazquez Acosta, T. Virdee, S. Wakefield, N. Wardle, T. Whyntie, M. Chadwick, J. E. Cole, P. R. Hobson, A. Khan, P. Kyberd, D. Leggat, D. Leslie, W. Martin, I. D. Reid, P. Symonds, L. Teodorescu, M. Turner, K. Hatakeyama, H. Liu, T. Scarborough, O. Charaf, C. Henderson, P. Rumerio, A. Avetisyan, T. Bose, C. Fantasia, A. Heister, J. St. John, P. Lawson, D. Lazic, J. Rohlf, D. Sperka, L. Sulak, J. Alimena, S. Bhattacharya, D. Cutts, Z. Demiragli, A. Ferapontov, A. Garabedian, U. Heintz, S. Jabeen, G. Kukartsev, E. Laird, G. Landsberg, M. Luk, M. Narain, D. Nguyen, M. Segala, T. Sinthuprasith, T. Speer, K. V. Tsang, R. Breedon, G. Breto, M. Calderon De La Barca Sanchez, S. Chauhan, M. Chertok, J. Conway, R. Conway, P. T. Cox, J. Dolen, R. Erbacher, M. Gardner, R. Houtz, W. Ko, A. Kopecky, R. Lander, O. Mall, T. Miceli, D. Pellett, F. Ricci-Tam, B. Rutherford, M. Searle, J. Smith, M. Squires, M. Tripathi, R. Vasquez Sierra, R. Yohay, V. Andreev, D. Cline, R. Cousins, J. Duris, S. Erhan, P. Everaerts, C. Farrell, J. Hauser, M. Ignatenko, C. Jarvis, C. Plager, G. Rakness, P. Schlein, P. Traczyk, V. Valuev, M. Weber, J. Babb, R. Clare, M. E. Dinardo, J. Ellison, J. W. Gary, F. Giordano, G. Hanson, G. Y. Jeng, H. Liu, O. R. Long, A. Luthra, H. Nguyen, S. Paramesvaran, J. Sturdy, S. Sumowidagdo, R. Wilken, S. Wimpenny, W. Andrews, J. G. Branson, G. B. Cerati, S. Cittolin, D. Evans, F. Golf, A. Holzner, R. Kelley, M. Lebourgeois, J. Letts, I. Macneill, B. Mangano, S. Padhi, C. Palmer, G. Petrucciani, M. Pieri, M. Sani, V. Sharma, S. Simon, E. Sudano, M. Tadel, Y. Tu, A. Vartak, S. Wasserbaech, F. Würthwein, A. Yagil, J. Yoo, D. Barge, R. Bellan, C. Campagnari, M. D’Alfonso, T. Danielson, K. Flowers, P. Geffert, J. Incandela, C. Justus, P. Kalavase, S. A. Koay, D. Kovalskyi, V. Krutelyov, S. Lowette, N. Mccoll, V. Pavlunin, F. Rebassoo, J. Ribnik, J. Richman, R. Rossin, D. Stuart, W. To, C. West, A. Apresyan, A. Bornheim, Y. Chen, E. Di Marco, J. Duarte, M. Gataullin, Y. Ma, A. Mott, H. B. Newman, C. Rogan, M. Spiropulu, V. Timciuc, J. Veverka, R. Wilkinson, S. Xie, Y. Yang, R. Y. Zhu, B. Akgun, V. Azzolini, A. Calamba, R. Carroll, T. Ferguson, Y. Iiyama, D. W. Jang, Y. F. Liu, M. Paulini, H. Vogel, I. Vorobiev, J. P. Cumalat, B. R. Drell, W. T. Ford, A. Gaz, E. Luiggi Lopez, J. G. Smith, K. Stenson, K. A. Ulmer, S. R. Wagner, J. Alexander, A. Chatterjee, N. Eggert, L. K. Gibbons, B. Heltsley, A. Khukhunaishvili, B. Kreis, N. Mirman, G. Nicolas Kaufman, J. R. Patterson, A. Ryd, E. Salvati, W. Sun, W. D. Teo, J. Thom, J. Thompson, J. Tucker, J. Vaughan, Y. Weng, L. Winstrom, P. Wittich, D. Winn, S. Abdullin, M. Albrow, J. Anderson, L. A. T. Bauerdick, A. Beretvas, J. Berryhill, P. C. Bhat, I. Bloch, K. Burkett, J. N. Butler, V. Chetluru, H. W. K. Cheung, F. Chlebana, V. D. Elvira, I. Fisk, J. Freeman, Y. Gao, D. Green, O. Gutsche, J. Hanlon, R. M. Harris, J. Hirschauer, B. Hooberman, S. Jindariani, M. Johnson, U. Joshi, B. Kilminster, B. Klima, S. Kunori, S. Kwan, C. Leonidopoulos, J. Linacre, D. Lincoln, R. Lipton, J. Lykken, K. Maeshima, J. M. Marraffino, S. Maruyama, D. Mason, P. McBride, K. Mishra, S. Mrenna, Y. Musienko, C. Newman-Holmes, V. O’Dell, O. Prokofyev, E. Sexton-Kennedy, S. Sharma, W. J. Spalding, L. Spiegel, L. Taylor, S. Tkaczyk, N. V. Tran, L. Uplegger, E. W. Vaandering, R. Vidal, J. Whitmore, W. Wu, F. Yang, F. Yumiceva, J. C. Yun, D. Acosta, P. Avery, D. Bourilkov, M. Chen, T. Cheng, S. Das, M. De Gruttola, G. P. Di Giovanni, D. Dobur, A. Drozdetskiy, R. D. Field, M. Fisher, Y. Fu, I. K. Furic, J. Gartner, J. Hugon, B. Kim, J. Konigsberg, A. Korytov, A. Kropivnitskaya, T. Kypreos, J. F. Low, K. Matchev, P. Milenovic, G. Mitselmakher, L. Muniz, M. Park, R. Remington, A. Rinkevicius, P. Sellers, N. Skhirtladze, M. Snowball, J. Yelton, M. Zakaria, V. Gaultney, S. Hewamanage, L. M. Lebolo, S. Linn, P. Markowitz, G. Martinez, J. L. Rodriguez, T. Adams, A. Askew, J. Bochenek, J. Chen, B. Diamond, S. V. Gleyzer, J. Haas, S. Hagopian, V. Hagopian, M. Jenkins, K. F. Johnson, H. Prosper, V. Veeraraghavan, M. Weinberg, M. M. Baarmand, B. Dorney, M. Hohlmann, H. Kalakhety, I. Vodopiyanov, M. R. Adams, I. M. Anghel, L. Apanasevich, Y. Bai, V. E. Bazterra, R. R. Betts, I. Bucinskaite, J. Callner, R. Cavanaugh, O. Evdokimov, L. Gauthier, C. E. Gerber, D. J. Hofman, S. Khalatyan, F. Lacroix, M. Malek, C. O’Brien, C. Silkworth, D. Strom, P. Turner, N. Varelas, U. Akgun, E. A. Albayrak, B. Bilki, W. Clarida, F. Duru, J.-P. Merlo, H. Mermerkaya, A. Mestvirishvili, A. Moeller, J. Nachtman, C. R. Newsom, E. Norbeck, Y. Onel, F. Ozok, S. Sen, P. Tan, E. Tiras, J. Wetzel, T. Yetkin, K. Yi, B. A. Barnett, B. Blumenfeld, S. Bolognesi, D. Fehling, G. Giurgiu, A. V. Gritsan, Z. J. Guo, G. Hu, P. Maksimovic, S. Rappoccio, M. Swartz, A. Whitbeck, P. Baringer, A. Bean, G. Benelli, R. P. Kenny Iii, M. Murray, D. Noonan, S. Sanders, R. Stringer, G. Tinti, J. S. Wood, V. Zhukova, A. F. Barfuss, T. Bolton, I. Chakaberia, A. Ivanov, S. Khalil, M. Makouski, Y. Maravin, S. Shrestha, I. Svintradze, J. Gronberg, D. Lange, D. Wright, A. Baden, M. Boutemeur, B. Calvert, S. C. Eno, J. A. Gomez, N. J. Hadley, R. G. Kellogg, M. Kirn, T. Kolberg, Y. Lu, M. Marionneau, A. C. Mignerey, K. Pedro, A. Skuja, J. Temple, M. B. Tonjes, S. C. Tonwar, E. Twedt, A. Apyan, G. Bauer, J. Bendavid, W. Busza, E. Butz, I. A. Cali, M. Chan, V. Dutta, G. Gomez Ceballos, M. Goncharov, K. A. Hahn, Y. Kim, M. Klute, K. Krajczar, P. D. Luckey, T. Ma, S. Nahn, C. Paus, D. Ralph, C. Roland, G. Roland, M. Rudolph, G. S. F. Stephans, F. Stöckli, K. Sumorok, K. Sung, D. Velicanu, E. A. Wenger, R. Wolf, B. Wyslouch, M. Yang, Y. Yilmaz, A. S. Yoon, M. Zanetti, S. I. Cooper, B. Dahmes, A. De Benedetti, G. Franzoni, A. Gude, S. C. Kao, K. Klapoetke, Y. Kubota, J. Mans, N. Pastika, R. Rusack, M. Sasseville, A. Singovsky, N. Tambe, J. Turkewitz, L. M. Cremaldi, R. Kroeger, L. Perera, R. Rahmat, D. A. Sanders, E. Avdeeva, K. Bloom, S. Bose, D. R. Claes, A. Dominguez, M. Eads, J. Keller, I. Kravchenko, J. Lazo-Flores, H. Malbouisson, S. Malik, G. R. Snow, A. Godshalk, I. Iashvili, S. Jain, A. Kharchilava, A. Kumar, G. Alverson, E. Barberis, D. Baumgartel, M. Chasco, J. Haley, D. Nash, D. Trocino, D. Wood, J. Zhang, A. Anastassov, A. Kubik, L. Lusito, N. Mucia, N. Odell, R. A. Ofierzynski, B. Pollack, A. Pozdnyakov, M. Schmitt, S. Stoynev, M. Velasco, S. Won, L. Antonelli, D. Berry, A. Brinkerhoff, K. M. Chan, M. Hildreth, C. Jessop, D. J. Karmgard, J. Kolb, K. Lannon, W. Luo, S. Lynch, N. Marinelli, D. M. Morse, T. Pearson, M. Planer, R. Ruchti, J. Slaunwhite, N. Valls, M. Wayne, M. Wolf, B. Bylsma, L. S. Durkin, C. Hill, R. Hughes, K. Kotov, T. Y. Ling, D. Puigh, M. Rodenburg, C. Vuosalo, G. Williams, B. L. Winer, N. Adam, E. Berry, P. Elmer, D. Gerbaudo, V. Halyo, P. Hebda, J. Hegeman, A. Hunt, P. Jindal, D. Lopes Pegna, P. Lujan, D. Marlow, T. Medvedeva, M. Mooney, J. Olsen, P. Piroué, X. Quan, A. Raval, B. Safdi, H. Saka, D. Stickland, C. Tully, J. S. Werner, A. Zuranski, E. Brownson, A. Lopez, H. Mendez, J. E. Ramirez Vargas, E. Alagoz, V. E. Barnes, D. Benedetti, G. Bolla, D. Bortoletto, M. De Mattia, A. Everett, Z. Hu, M. Jones, O. Koybasi, M. Kress, A. T. Laasanen, N. Leonardo, V. Maroussov, P. Merkel, D. H. Miller, N. Neumeister, I. Shipsey, D. Silvers, A. Svyatkovskiy, M. Vidal Marono, H. D. Yoo, J. Zablocki, Y. Zheng, S. Guragain, N. Parashar, A. Adair, C. Boulahouache, K. M. Ecklund, F. J. M. Geurts, W. Li, B. P. Padley, R. Redjimi, J. Roberts, J. Zabel, B. Betchart, A. Bodek, Y. S. Chung, R. Covarelli, P. de Barbaro, R. Demina, Y. Eshaq, T. Ferbel, A. Garcia-Bellido, P. Goldenzweig, J. Han, A. Harel, D. C. Miner, D. Vishnevskiy, M. Zielinski, A. Bhatti, R. Ciesielski, L. Demortier, K. Goulianos, G. Lungu, S. Malik, C. Mesropian, S. Arora, A. Barker, J. P. Chou, C. Contreras-Campana, E. Contreras-Campana, D. Duggan, D. Ferencek, Y. Gershtein, R. Gray, E. Halkiadakis, D. Hidas, A. Lath, S. Panwalkar, M. Park, R. Patel, V. Rekovic, J. Robles, K. Rose, S. Salur, S. Schnetzer, C. Seitz, S. Somalwar, R. Stone, S. Thomas, M. Walker, G. Cerizza, M. Hollingsworth, S. Spanier, Z. C. Yang, A. York, R. Eusebi, W. Flanagan, J. Gilmore, T. Kamon, V. Khotilovich, R. Montalvo, I. Osipenkov, Y. Pakhotin, A. Perloff, J. Roe, A. Safonov, T. Sakuma, S. Sengupta, I. Suarez, A. Tatarinov, D. Toback, N. Akchurin, J. Damgov, C. Dragoiu, P. R. Dudero, C. Jeong, K. Kovitanggoon, S. W. Lee, T. Libeiro, Y. Roh, I. Volobouev, E. Appelt, A. G. Delannoy, C. Florez, S. Greene, A. Gurrola, W. Johns, P. Kurt, C. Maguire, A. Melo, M. Sharma, P. Sheldon, B. Snook, S. Tuo, J. Velkovska, M. W. Arenton, M. Balazs, S. Boutle, B. Cox, B. Francis, J. Goodell, R. Hirosky, A. Ledovskoy, C. Lin, C. Neu, J. Wood, S. Gollapinni, R. Harr, P. E. Karchin, C. Kottachchi Kankanamge Don, P. Lamichhane, A. Sakharov, M. Anderson, D. A. Belknap, L. Borrello, D. Carlsmith, M. Cepeda, S. Dasu, E. Friis, L. Gray, K. S. Grogg, M. Grothe, R. Hall-Wilton, M. Herndon, A. Hervé, P. Klabbers, J. Klukas, A. Lanaro, C. Lazaridis, J. Leonard, R. Loveless, A. Mohapatra, I. Ojalvo, F. Palmonari, G. A. Pierro, I. Ross, A. Savin, W. H. Smith, J. Swanson

**Affiliations:** 1 CERN, Geneva, Switzerland; 2 Yerevan Physics Institute, Yerevan, Armenia; 3 Institut für Hochenergiephysik der OeAW, Wien, Austria; 4 National Centre for Particle and High Energy Physics, Minsk, Belarus; 5 Universiteit Antwerpen, Antwerpen, Belgium; 6 Vrije Universiteit Brussel, Brussel, Belgium; 7 Université Libre de Bruxelles, Bruxelles, Belgium; 8 Ghent University, Ghent, Belgium; 9 Université Catholique de Louvain, Louvain-la-Neuve, Belgium; 10 Université de Mons, Mons, Belgium; 11 Centro Brasileiro de Pesquisas Fisicas, Rio de Janeiro, Brazil; 12 Universidade do Estado do Rio de Janeiro, Rio de Janeiro, Brazil; 13 Universidade Estadual Paulista, São Paulo, Brazil; 14 Universidade Federal do ABC, São Paulo, Brazil; 15 Institute for Nuclear Research and Nuclear Energy, Sofia, Bulgaria; 16 University of Sofia, Sofia, Bulgaria; 17 Institute of High Energy Physics, Beijing, China; 18 State Key Lab. of Nucl. Phys. and Tech., Peking University, Beijing, China; 19 Universidad de Los Andes, Bogota, Colombia; 20 Technical University of Split, Split, Croatia; 21 University of Split, Split, Croatia; 22 Institute Rudjer Boskovic, Zagreb, Croatia; 23 University of Cyprus, Nicosia, Cyprus; 24 Charles University, Prague, Czech Republic; 25 Egyptian Network of High Energy Physics, Academy of Scientific Research and Technology of the Arab Republic of Egypt, Cairo, Egypt; 26 National Institute of Chemical Physics and Biophysics, Tallinn, Estonia; 27 Department of Physics, University of Helsinki, Helsinki, Finland; 28 Helsinki Institute of Physics, Helsinki, Finland; 29 Lappeenranta University of Technology, Lappeenranta, Finland; 30 DSM/IRFU, CEA/Saclay, Gif-sur-Yvette, France; 31 Laboratoire Leprince-Ringuet, Ecole Polytechnique, IN2P3-CNRS, Palaiseau, France; 32 Institut Pluridisciplinaire Hubert Curien, Université de Strasbourg, Université de Haute Alsace Mulhouse, CNRS/IN2P3, Strasbourg, France; 33 Centre de Calcul de l’Institut National de Physique Nucleaire et de Physique des Particules, CNRS/IN2P3, Villeurbanne, France; 34 Université de Lyon, Université Claude Bernard Lyon 1 CNRS-IN2P3, Institut de Physique Nucléaire de Lyon, Villeurbanne, France; 35 Institute of High Energy Physics and Informatization, Tbilisi State University, Tbilisi, Georgia; 36 I. Physikalisches Institut, RWTH Aachen University, Aachen, Germany; 37 III. Physikalisches Institut A, RWTH Aachen University, Aachen, Germany; 38 III. Physikalisches Institut B, RWTH Aachen University, Aachen, Germany; 39 Deutsches Elektronen-Synchrotron, Hamburg, Germany; 40 University of Hamburg, Hamburg, Germany; 41 Institut für Experimentelle Kernphysik, Karlsruhe, Germany; 42 Institute of Nuclear Physics “Demokritos”, Aghia Paraskevi, Greece; 43 University of Athens, Athens, Greece; 44 University of Ioánnina, Ioánnina, Greece; 45 KFKI Research Institute for Particle and Nuclear Physics, Budapest, Hungary; 46 Institute of Nuclear Research ATOMKI, Debrecen, Hungary; 47 University of Debrecen, Debrecen, Hungary; 48 Panjab University, Chandigarh, India; 49 University of Delhi, Delhi, India; 50 Saha Institute of Nuclear Physics, Kolkata, India; 51 Bhabha Atomic Research Centre, Mumbai, India; 52 Tata Institute of Fundamental Research - EHEP, Mumbai, India; 53 Tata Institute of Fundamental Research - HECR, Mumbai, India; 54 Institute for Research in Fundamental Sciences (IPM), Tehran, Iran; 55 INFN Sezione di Bari, Bari, Italy; 56 Università di Bari, Bari, Italy; 57 Politecnico di Bari, Bari, Italy; 58 INFN Sezione di Bologna, Bologna, Italy; 59 Università di Bologna, Bologna, Italy; 60 INFN Sezione di Catania, Catania, Italy; 61 Università di Catania, Catania, Italy; 62 INFN Sezione di Firenze, Firenze, Italy; 63 Università di Firenze, Firenze, Italy; 64 INFN Laboratori Nazionali di Frascati, Frascati, Italy; 65 INFN Sezione di Genova, Genova, Italy; 66 Università di Genova, Genova, Italy; 67 INFN Sezione di Milano-Bicocca, Milano, Italy; 68 Università di Milano-Bicocca, Milano, Italy; 69 INFN Sezione di Napoli, Napoli, Italy; 70 Università di Napoli ’Federico II’, Napoli, Italy; 71 Università della Basilicata (Potenza), Napoli, Italy; 72 Università G. Marconi (Roma), Napoli, Italy; 73 INFN Sezione di Padova, Padova, Italy; 74 Università di Padova, Padova, Italy; 75 Università di Trento (Trento), Padova, Italy; 76 INFN Sezione di Pavia, Pavia, Italy; 77 Università di Pavia, Pavia, Italy; 78 INFN Sezione di Perugia, Perugia, Italy; 79 Università di Perugia, Perugia, Italy; 80 INFN Sezione di Pisa, Pisa, Italy; 81 Università di Pisa, Pisa, Italy; 82 Scuola Normale Superiore di Pisa, Pisa, Italy; 83 INFN Sezione di Roma, Roma, Italy; 84 Università di Roma, Roma, Italy; 85 INFN Sezione di Torino, Torino, Italy; 86 Università di Torino, Torino, Italy; 87 Università del Piemonte Orientale (Novara), Torino, Italy; 88 INFN Sezione di Trieste, Trieste, Italy; 89 Università di Trieste, Trieste, Italy; 90 Kangwon National University, Chunchon, Korea; 91 Kyungpook National University, Daegu, Korea; 92 Institute for Universe and Elementary Particles, Chonnam National University, Kwangju, Korea; 93 Korea University, Seoul, Korea; 94 University of Seoul, Seoul, Korea; 95 Sungkyunkwan University, Suwon, Korea; 96 Vilnius University, Vilnius, Lithuania; 97 Centro de Investigacion y de Estudios Avanzados del IPN, Mexico City, Mexico; 98 Universidad Iberoamericana, Mexico City, Mexico; 99 Benemerita Universidad Autonoma de Puebla, Puebla, Mexico; 100 Universidad Autónoma de San Luis Potosí, San Luis Potosí, Mexico; 101 University of Auckland, Auckland, New Zealand; 102 University of Canterbury, Christchurch, New Zealand; 103 National Centre for Physics, Quaid-I-Azam University, Islamabad, Pakistan; 104 National Centre for Nuclear Research, Swierk, Poland; 105 Institute of Experimental Physics, Faculty of Physics, University of Warsaw, Warsaw, Poland; 106 Laboratório de Instrumentação e Física Experimental de Partículas, Lisboa, Portugal; 107 Joint Institute for Nuclear Research, Dubna, Russia; 108 Petersburg Nuclear Physics Institute, Gatchina (St. Petersburg), Russia; 109 Institute for Nuclear Research, Moscow, Russia; 110 Institute for Theoretical and Experimental Physics, Moscow, Russia; 111 Moscow State University, Moscow, Russia; 112 P.N. Lebedev Physical Institute, Moscow, Russia; 113 State Research Center of Russian Federation, Institute for High Energy Physics, Protvino, Russia; 114 Faculty of Physics and Vinca Institute of Nuclear Sciences, University of Belgrade, Belgrade, Serbia; 115 Centro de Investigaciones Energéticas Medioambientales y Tecnológicas (CIEMAT), Madrid, Spain; 116 Universidad Autónoma de Madrid, Madrid, Spain; 117 Universidad de Oviedo, Oviedo, Spain; 118 Instituto de Física de Cantabria (IFCA), CSIC-Universidad de Cantabria, Santander, Spain; 119 CERN, European Organization for Nuclear Research, Geneva, Switzerland; 120 Paul Scherrer Institut, Villigen, Switzerland; 121 Institute for Particle Physics, ETH Zurich, Zurich, Switzerland; 122 Universität Zürich, Zurich, Switzerland; 123 National Central University, Chung-Li, Taiwan; 124 National Taiwan University (NTU), Taipei, Taiwan; 125 Chulalongkorn University, Bangkok, Thailand; 126 Cukurova University, Adana, Turkey; 127 Physics Department, Middle East Technical University, Ankara, Turkey; 128 Bogazici University, Istanbul, Turkey; 129 Istanbul Technical University, Istanbul, Turkey; 130 National Scientific Center, Kharkov Institute of Physics and Technology, Kharkov, Ukraine; 131 University of Bristol, Bristol, United Kingdom; 132 Rutherford Appleton Laboratory, Didcot, United Kingdom; 133 Imperial College, London, United Kingdom; 134 Brunel University, Uxbridge, United Kingdom; 135 Baylor University, Waco, USA; 136 The University of Alabama, Tuscaloosa, USA; 137 Boston University, Boston, USA; 138 Brown University, Providence, USA; 139 University of California, Davis, Davis, USA; 140 University of California, Los Angeles, USA; 141 University of California, Riverside, Riverside, USA; 142 University of California, San Diego, La Jolla, USA; 143 University of California, Santa Barbara, Santa Barbara, USA; 144 California Institute of Technology, Pasadena, USA; 145 Carnegie Mellon University, Pittsburgh, USA; 146 University of Colorado at Boulder, Boulder, USA; 147 Cornell University, Ithaca, USA; 148 Fairfield University, Fairfield, USA; 149 Fermi National Accelerator Laboratory, Batavia, USA; 150 University of Florida, Gainesville, USA; 151 Florida International University, Miami, USA; 152 Florida State University, Tallahassee, USA; 153 Florida Institute of Technology, Melbourne, USA; 154 University of Illinois at Chicago (UIC), Chicago, USA; 155 The University of Iowa, Iowa City, USA; 156 Johns Hopkins University, Baltimore, USA; 157 The University of Kansas, Lawrence, USA; 158 Kansas State University, Manhattan, USA; 159 Lawrence Livermore National Laboratory, Livermore, USA; 160 University of Maryland, College Park, USA; 161 Massachusetts Institute of Technology, Cambridge, USA; 162 University of Minnesota, Minneapolis, USA; 163 University of Mississippi, Oxford, USA; 164 University of Nebraska-Lincoln, Lincoln, USA; 165 State University of New York at Buffalo, Buffalo, USA; 166 Northeastern University, Boston, USA; 167 Northwestern University, Evanston, USA; 168 University of Notre Dame, Notre Dame, USA; 169 The Ohio State University, Columbus, USA; 170 Princeton University, Princeton, USA; 171 University of Puerto Rico, Mayaguez, USA; 172 Purdue University, West Lafayette, USA; 173 Purdue University Calumet, Hammond, USA; 174 Rice University, Houston, USA; 175 University of Rochester, Rochester, USA; 176 The Rockefeller University, New York, USA; 177 Rutgers, the State University of New Jersey, Piscataway, USA; 178 University of Tennessee, Knoxville, USA; 179 Texas A&M University, College Station, USA; 180 Texas Tech University, Lubbock, USA; 181 Vanderbilt University, Nashville, USA; 182 University of Virginia, Charlottesville, USA; 183 Wayne State University, Detroit, USA; 184 University of Wisconsin, Madison, USA

## Abstract

Results are reported from a search for new physics processes in events containing a single isolated high-transverse-momentum lepton (electron or muon), energetic jets, and large missing transverse momentum. The analysis is based on a 4.98 fb^−1^ sample of proton–proton collisions at a center-of-mass energy of 7 TeV, obtained with the CMS detector at the LHC. Three separate background estimation methods, each relying primarily on control samples in the data, are applied to a range of signal regions, providing complementary approaches for estimating the background yields. The observed yields are consistent with the predicted standard model backgrounds. The results are interpreted in terms of limits on the parameter space for the constrained minimal supersymmetric extension of the standard model, as well as on cross sections for simplified models, which provide a generic description of the production and decay of new particles in specific, topology based final states.

**Electronic Supplementary Material:**

The online version of this article (doi:10.1140/epjc/s10052-013-2404-z) contains supplementary material, which is available to authorized users.

## Introduction

This paper reports results from an updated and improved search for new physics processes in proton–proton collisions at a center-of-mass energy of 7 TeV, focusing on the signature with a single isolated lepton (electron or muon), multiple energetic jets, and large missing momentum transverse to the beam direction (). The data sample was collected by the Compact Muon Solenoid (CMS) experiment during 2011 at the Large Hadron Collider (LHC) and corresponds to an integrated luminosity of 4.98 fb^−1^, roughly one hundred times larger than the sample used for our previous search [1].

The  signature is prominent in models based on supersymmetry (SUSY) [2–7]. In *R*-parity-conserving models [8], SUSY particles are produced in pairs, and their decay chains end with the lightest supersymmetric particle (LSP). In some scenarios, the LSP is a neutralino ($\widetilde{\chi}^{0}$), a heavy, electrically neutral, weakly interacting particle with the properties of a dark-matter candidate [9]. The presence of two such LSPs in each SUSY event typically leads to a large missing transverse momentum, depending on the details of the SUSY mass spectrum. The isolated lepton indicates a weak decay of a heavy particle, such as a W boson or a chargino ($\widetilde{\chi}^{\pm}$). Multiple jets can be produced in complex decay chains of SUSY particles. This signature arises in many SUSY models, including the constrained minimal supersymmetric extension of the standard model (CMSSM) [10, 11], and in simplified models [12–15], which are based on simplified mass spectra and decays of new particles. Both of these frameworks are used to interpret the results. Searches in this or similar channels have been reported by CMS [1, 16] and ATLAS [17–19].

Searches for SUSY particles are complicated by the presence of standard model (SM) backgrounds that can share many of the features of signal events. In the single-lepton final state, backgrounds arise primarily from the production of $\mathrm{t}\overline{\mathrm{t}}$ and W+jets events, with smaller contributions from Z+jets, single-top quark production, and QCD multijet events. In the event topology studied here, a large observed value of  in a standard model event is usually genuine, resulting from the production of one or more high-momentum neutrinos. A smaller contribution to events in the high- tail in this search can arise from the mismeasurement of jets in high cross section processes such as QCD multijet events. To determine the contributions from these backgrounds, we use methods that are primarily based on control samples in data, sometimes in conjunction with specific information from simulated event samples or from additional measurements that provide constraints on the background processes.

Three complementary methods are used to analyze the data, providing valuable cross-checks and probing different signal regions. The *Lepton Spectrum* (*LS*) *method* was used in the CMS single-lepton [1] and opposite-sign dilepton [20] SUSY searches performed using the 2010 data sample. It uses the observed lepton transverse momentum (*p*_T_) spectrum and other control samples to predict the  distribution associated with the dominant SM backgrounds. This method is sensitive to SUSY models in which the  distribution is decoupled from the lepton *p*_T_ spectrum, as is the case when two undetected LSPs produce a large missing transverse momentum. The *Lepton-Projection Variable* (*L*_P_) *method* uses the *L*_P_ variable, which was developed for the CMS measurement of the W polarization in W+jets events [21]. This variable, described in Sect. 6, is correlated with the helicity angle of the lepton in the W-boson rest frame. Both the *L*_P_ and the LS methods take advantage of well-understood properties of the W polarization in $\mathrm{t}\overline{\mathrm{t}}$ and W+jets events for the background determination. The methods are complementary in that they rely on significantly different approaches to determining the backgrounds, based on different kinematic variables and different signal regions. The *ANN method* uses an artificial neural network discriminant built from several kinematic quantities. The ANN discriminant is then used in conjunction with  to define signal and sideband regions, from which the background yield is determined. A key variable in the ANN is *M*_T_, an approximate invariant mass of the system comprising the lepton and the , computed with the momentum components transverse to the beam direction. Background events usually have *M*_T_<*M*(W), where *M*(W) is the W boson mass, because the observed  is associated with the neutrino from $\mathrm{W}\to\ell\bar{\nu}$ decay.

This paper is organized as follows. Sections 2 and 3 describe the CMS detector and the event samples. The event preselection requirements that are common to all methods are discussed in Sect. 4. Sections 5, 6, and 7 describe the LS, *L*_P_, and ANN methods, respectively, for obtaining SM background estimates from control samples in data. The observed yields in data are compared with the background estimate obtained for each method. Systematic uncertainties are described in Sect. 8. Finally, the results, interpretation, and conclusions of the analysis are presented in Sects. 9 and 10.

## The CMS detector

The CMS detector, described in detail in Ref. [22], is a multipurpose apparatus designed to study high-*p*_T_ physics processes in proton–proton collisions, as well as a broad range of phenomena in heavy-ion collisions. The central element of CMS is a 3.8 T superconducting solenoid, 13 m in length and 6 m in diameter. Within the magnet are (in order of increasing distance from the beam pipe) high-precision silicon pixel and silicon strip detectors for charged particle tracking; a lead–tungstate crystal electromagnetic calorimeter for measurements of photons, electrons, and the electromagnetic component of jets; and a hadron calorimeter, constructed from scintillating tiles and brass absorbers, for jet energy measurements. Beyond the magnet is the muon system, comprising drift tube, cathode strip, and resistive-plate detectors interleaved with steel absorbers. Most of the detector systems are divided into subsystems that cover the central (barrel) and forward (endcap) regions. The first level of the CMS trigger consists of custom hardware processors that use information from the calorimeter and the muon system to select up to 100 kHz of the most interesting events. These events are then analyzed in the High Level Trigger (HLT) processor farm, which uses information from all CMS detector systems to reduce the event rate to about 300 Hz.

In describing the angular distribution of particles and the acceptance of the detector, we frequently make use of the pseudorapidity, *η*=−ln[tan(*θ*/2)], where the polar angle *θ* of the particle’s momentum vector is measured with respect to the *z* axis of the CMS coordinate system. The *z* axis points along the direction of the counterclockwise-moving proton beam; the azimuthal angle *ϕ* is measured in a plane perpendicular to this axis. The separation between two momentum vectors in *η*–*ϕ* space is characterized by the quantity $\Delta R = \sqrt{(\Delta\eta)^{2}+ (\Delta\phi)^{2}}$, which is approximately invariant under Lorentz boosts along the *z* axis.

## Data and simulated event samples

The data samples used in the analysis were selected using triggers based on , lepton *p*_T_, and the transverse momenta ($p_{\mathrm{T}} ^{j}$) of the observed jets *j*. The overall level of jet activity was measured with the quantity $H_{\mathrm{T}}^{\mathrm{trigger}}=\sum_{j} p_{\mathrm{T}}^{j}$, the scalar sum of jet transverse momenta satisfying $p_{\mathrm{T}}^{j}>40~\mathrm{GeV}$. The missing transverse momentum  was computed in the trigger using particle-flow algorithms [23, 24]. To maintain an acceptable trigger rate, the thresholds on , lepton *p*_T_, and $H_{\mathrm{T}} ^{\mathrm{trigger}}$, were raised as the LHC luminosity increased over the course of the data collection period. The highest thresholds applied in the muon trigger selection were , muon *p*_T_>15 GeV, and $H_{\mathrm{T}}^{\mathrm{trigger}}>300~\mathrm{GeV}$. For electron triggers, the highest thresholds applied were , electron *p*_T_>15 GeV and $H_{\mathrm{T}}^{\mathrm{trigger}}> 250~\mathrm{GeV}$; a loose electron isolation requirement was also applied to help control the rate. The offline analysis requirements for both muon and electron events are more restrictive than those used in the trigger.

The analysis procedures are designed using simulated event samples. Except for certain scans of the SUSY parameter space discussed later, the detector simulation is performed using the Geant4 package [25]. A variety of Monte Carlo (MC) event generators are used to model the backgrounds. The QCD multijet samples are generated with the pythia 6.4.22 [26] MC generator with tune Z2 [27]. The dominant background, $\mathrm{t}\overline{\mathrm{t}}$, is studied with a sample generated using MadGraph 5.1.1.0 [28]. The W+jets and Z+jets processes are also simulated with MadGraph. Single-top (*s*-channel, *t*-channel, and tW) production is simulated with powheg [29]. To model the effect of multiple pp interactions per beam crossing (pileup), simulated events are generated with a nominal distribution of multiple vertices, then reweighted to match the distribution of the number of collision vertices per bunch crossing as measured in data.

Event samples for SUSY benchmark models are generated with pythia. As example CMSSM scenarios, we use LM3 and LM6, which are among the standard benchmarks [30] used in CMS. The CMSSM benchmarks are described by the universal scalar mass parameter *m*_0_, the universal gaugino mass parameter *m*_1/2_, the universal trilinear soft-SUSY-breaking parameter *A*_0_, the ratio of the two Higgs-doublet vacuum expectation values tan*β*, and the sign of the Higgs mixing parameter *μ*. The LM3 (LM6) benchmark is described by *m*_0_=330 GeV (85 GeV), *m*_1/2_=240 GeV (400 GeV), *A*_0_=0 GeV (0 GeV), tan*β*=20 (10), and *μ*>0 (0). For LM3, the masses of the gluino and squarks are very similar (≈600 GeV), except for $m(\widetilde{\mathrm{t}})\approx440~\mathrm{GeV}$, while the mass of the LSP is $m(\widetilde{\chi}^{0}_{1}) = 94~\mathrm{GeV}$. The LM6 spectrum is heavier, with $m(\widetilde{\mathrm{g}})\approx930~\mathrm{GeV}$, $m(\widetilde {\mathrm{q}})\approx800~\mathrm{GeV}$, $m(\widetilde{\mathrm{t}})\approx650~\mathrm{GeV}$, and $m(\widetilde{\chi }^{0}_{1})\approx 160~\mathrm{GeV}$. The next-to-leading-order (NLO) cross sections for these models are approximately 4.8 pb (LM3), and 0.4 pb (LM6).

The ANN method uses the LM0 model [30] to train the neural network. Because of its large cross section (54.9 pb at NLO), LM0 has already been excluded [1], but its kinematic distributions still provide a reasonably generic description of SUSY behavior with respect to the variables used in the neural network. The parameters for LM0 are *m*_0_=200 GeV, *m*_1/2_=160 GeV, *A*_0_=−400 GeV, tan*β*=10, and *μ*>0.

The results are interpreted in two ways: (i) as constraints on CMSSM parameter space and (ii) as constraints on cross sections for event topologies described in the framework of simplified models. In both cases, a large number of simulated event samples are required to scan over the relevant space of model parameters. For this reason, the scans are performed with the CMS fast simulation package [31], which reduces the time associated with the detector simulation.

Both the LS and *L*_P_ background determination methods rely on knowledge of the W-boson polarization in W+jets and in $\mathrm{t}\overline {\mathrm{t}}$ events. The polarization effects are well modeled in simulated event samples, which are used in conjunction with control samples in data. The angular distribution of the (positively) charged lepton in the W^+^ rest frame can be written as: 1 where *f*_+1_, *f*_−1_, and *f*_0_ denote the polarization fractions associated with the W-boson helicities +1, −1, and 0, respectively. The angle $\theta^{*}_{\ell}$ is the polar angle of the charged lepton in the W^+^ rest frame, measured with respect to a *z* axis that is aligned with the momentum direction of the W^+^ in the top-quark rest frame. The polarization fractions thus determine the angular distribution of the lepton in the W rest frame and, together with the Lorentz boosts, control the *p*_T_ distributions of the lepton and the neutrino in the laboratory frame.

The W polarization fractions in top-quark decays have been calculated [32] with QCD corrections to next-to-next-to-leading order (NNLO), and the polarization is predominantly longitudinal. For t→bW^+^ these fractions are *f*_0_=0.687±0.005, *f*_−1_=0.311±0.005, and *f*_+1_=0.0017±0.0001. These precise calculations reduce the uncertainties associated with the W polarization in $\mathrm{t}\overline {\mathrm{t}}$ events to a low level. The theoretical values are consistent with measurements from ATLAS [33], which obtained *f*_0_=0.67±0.03±0.06, *f*_−1_=0.32±0.02±0.03, and *f*_+1_=0.01±0.01±0.04, expressed for the W^+^ polarizations.

The W polarization in W+jets events exhibits a more complex behavior than that in $\mathrm{t}\overline{\mathrm{t}}$ production. Both CMS [21] and ATLAS [34] have reported measurements of these effects, which are consistent with alpgen [35] and MadGraph [28] simulations predicting that the W^+^ and W^−^ bosons are both predominantly left-handed in W+jets events at high *p*_T_. An NLO QCD calculation [36] has demonstrated that the predicted polarization fractions are stable with respect to QCD corrections. As discussed in later sections, this detailed knowledge of the W-boson polarization provides key information for measuring the SM backgrounds using control samples in data.

## Event preselection

Table 1 summarizes the main variables and requirements used in the event preselection, which is designed to be simple and robust. Except where noted, a common set of preselection requirements is used by each of the three analysis methods. Events are required to have at least one good reconstructed primary vertex, at least three jets (*L*_P_ method and ANN method) or four jets (LS method), and exactly one isolated muon or exactly one isolated electron. These basic requirements select an event sample that is dominated by genuine, single-lepton events from SM processes.

**Table 1:** Main preselection requirements. The term lepton designates either an electron or a muon. Definitions of the quantities and further details are given in the text

Quantity	Requirement
Primary vertex position	*ρ*_PV_<2 cm, |*z*_PV_|<24 cm
Jet *p*_T_ threshold	>40 GeV
Jet *η* range	|*η*|<2.4
Number of jets	≥3 (*L*_P_ and ANN methods), ≥4 (LS method)
Lepton *p*_T_ threshold	>20 GeV
Muon *η* range	|*η*|<2.1
Muon isolation (relative)	<0.10
Electron *η* range	|*η*|<1.442, 1.56<|*η*|<2.4
Electron isolation (relative)	<0.07 (barrel), <0.06 (endcaps)
Lepton *p*_T_ thresh. for veto	>15 GeV

The primary vertex must satisfy a set of quality requirements, including |*z*_PV_|<24 cm and *ρ*_PV_<2 cm, where *z*_PV_ and *ρ*_PV_ are the longitudinal and transverse distances of the primary vertex with respect to the nominal interaction point in the CMS detector.

Jets are reconstructed offline using the anti-*k*_T_ clustering algorithm [37] with a distance parameter of 0.5. The particle four-vectors reconstructed by the CMS particle-flow algorithm [23, 24], are used as inputs to the jet clustering algorithm. The particle-flow algorithm combines information from all CMS sub-detectors to provide a complete list of long-lived particles in the event. Corrections based on simulation are applied to the jet energies to establish a uniform response across the detector and a first approximation to the absolute energy scale [38]. Additional jet energy corrections are applied to the data using measurements of energy balance in dijet and photon+jet control samples in data. These additional corrections take into account residual differences between the jet energy scale in data and simulation. The effect of pileup was significant during much of the data-taking period. Extra energy clustered into jets due to pileup is taken into account with an event-by-event correction to the jet momentum four-vectors. Jet candidates are required to satisfy quality criteria that suppress noise and spurious energy deposits in the calorimeters. The performance of jet reconstruction and the corrections are described in Ref. [38]. In this analysis, reconstructed jets are required to satisfy *p*_T_>40 GeV and |*η*|<2.4. The  vector is defined as the negative of the vector sum of the transverse momenta of all the particles reconstructed and identified by the particle-flow algorithm.

In the muon channel, the preselection requires a single muon candidate [39] satisfying *p*_T_(*μ*)>20 GeV and |*η*|<2.1. Several requirements are imposed on the elements that form the muon candidate. The reconstructed track must satisfy quality criteria related to the number of hits in the pixel, strip, and muon detectors, and it must have an impact parameter *d*_0_ in the transverse plane with respect to the beam spot satisfying |*d*_0_|<0.02 cm and an impact parameter *d*_*z*_ with respect to the primary vertex along the *z* direction satisfying |*d*_*z*_|<1.0 cm.

To suppress background in which the muon originates from a semileptonic decay of a hadron containing a bottom or charm quark, we require that the muon candidate be spatially isolated from other energy in the event. A cone of size Δ*R*=0.3 is constructed around the initial muon momentum direction in *η*–*ϕ* space. The muon combined isolation variable, *I* ^comb^=∑_Δ*R*<0.3_(*E*_T_+*p*_T_), is defined as the sum of the transverse energy *E*_T_ (as measured in the electromagnetic and hadron calorimeters) and the transverse momentum *p*_T_ (as measured in the silicon tracker) of all reconstructed objects within this cone, excluding the muon. This quantity is used to compute the combined isolation relative to the muon transverse momentum, $I^{\mathrm{comb}}_{\mathrm{rel}}=I^{\mathrm{comb}}/p_{\mathrm{T}}(\mu)$, which is required to satisfy $I^{\mathrm{comb}}_{\mathrm{rel}}<0.1$.

Electron candidates [40] are reconstructed by matching energy clusters in the ECAL with tracks in the silicon tracking system. Candidates must satisfy *p*_T_>20 GeV and |*η*|<2.4, excluding the barrel-endcap transition region (1.442<|*η*|<1.56). Quality and photon-conversion rejection requirements are also imposed. The relative isolation variable, defined in a manner similar to that in the muon channel, must satisfy $I^{\mathrm{comb}}_{\mathrm{rel}}<0.07$ in the barrel region and $I^{\mathrm{comb}}_{\mathrm{rel}}<0.06$ in the endcaps. The requirements on *d*_0_ and *d*_*z*_ are the same as those used in the muon channel.

The preselection requirements have a large effect on the sample composition. The lepton isolation requirement is critical for the rejection of QCD multijet processes, which have very large cross sections but are reduced to a low level by the isolation and the other preselection requirements. While many lepton candidates are produced in the semileptonic decays of hadrons containing b or c quarks, from *π* and K decays in flight, and from misidentification of hadrons, the vast majority of these candidates are either within or near hadronic jets. The background from W+jets events (primarily from W→e*ν* or W→*μν*, but also W→*τν*) is initially also very large. This contribution is heavily suppressed by the three- or four-jet requirement. Depending on the particular signal region, either $\mathrm{t}\overline{\mathrm{t}}$ or W+jets production emerges as the largest contribution to the background in the sample of events with moderate to large .

Events with a second isolated-lepton candidate satisfying the criteria listed in Table 1 are vetoed. This requirement not only suppresses SM background, but also minimizes the statistical overlap between the event sample used in this search and those used in multilepton searches. However, $\mathrm{t}\overline{\mathrm{t}}$ events with dileptons can still be present, and this contribution must be determined, particularly because the presence of two neutrinos in the decay chains can result in large values of . The background involving W→*τν* decays, both from $\mathrm{t}\overline {\mathrm{t}}$ events and from direct W production, must also be determined. To help suppress the dilepton background, the requirements on the veto leptons are somewhat looser than those on the signal lepton. For both muons and electrons, the *p*_T_ threshold is *p*_T_>15 GeV, the isolation requirement is $I^{\mathrm{comb}}_{\mathrm{rel}}<0.15$, and the impact parameter requirement is |*d*_0_|<0.1 cm (the *d*_*z*_ requirement is kept the same as for the signal lepton). In addition, some of the quality requirements for both the muon and electron are loosened.

Further event selection requirements are used in the individual background estimation methods described in Sects. 5, 6, and 7. The methods use the quantity *H*_T_, which is defined as the scalar sum of the transverse momenta of particle-flow jets *j* with $p_{\mathrm{T}}^{j}>40~\mathrm{GeV}$ and |*η* ^*j*^|<2.4, 2


The three background determination methods presented in the following three sections use different approaches to estimating the SM backgrounds using control samples in data. In Sect. 9, we compare the results of the different methods and make some observations about their features.

## Lepton Spectrum method

### Overview of the Lepton Spectrum method

This section describes the Lepton Spectrum (LS) method, which is named for the technique used to determine the dominant background source: genuine, single-lepton processes. Such processes account for about 75 % of the total SM background in the signal regions and arise primarily from $\mathrm{t}\overline{\mathrm{t}}$, single-top, and W+jets events. Their contribution to the  distribution is estimated by exploiting the fact that, when the lepton is produced in W-boson decay, the  distribution is fundamentally related to the lepton *p*_T_ spectrum, unlike the  for many SUSY models. A more detailed description of the Lepton Spectrum method is given in the references [1, 41].

Non-single-lepton backgrounds are also determined using control samples in the data. Such events arise mainly from (i) $\mathrm{t}\overline{\mathrm{t}}$ dilepton events, in which zero, one, or both of the leptons is a *τ* and (ii) $\mathrm{t}\overline{\mathrm{t}}$ and W+jets events with a single *τ*→(*μ*,e) decay. Background from QCD multijet events is expected from simulation to be very small. However, the uncertainties in such simulations are difficult to quantify, because the QCD multijet background in the phase space relevant to this analysis arises from extreme tails of processes with very large cross sections. We therefore use control samples in data to measure the QCD multijet background. Simulated event samples are used for the determination of the Z+jets contribution, which is estimated with sufficient precision to be below one event for most of the signal regions.

The signal regions are defined with three thresholds in *H*_T_ (*H*_T_≥500 GeV, *H*_T_≥750 GeV, and *H*_T_≥1000 GeV) and four bins in  (, , , and ).

### Estimation of single-lepton backgrounds

The physical foundation of the Lepton Spectrum method is that, when the lepton and neutrino are produced together in two-body W decay (either in $\mathrm{t}\overline{\mathrm{t}}$ or in W+jets events), the lepton *p*_T_ spectrum is directly related to the  spectrum. The lepton and the neutrino share a common Lorentz boost from the W rest frame to the laboratory frame. As a consequence, the lepton spectrum reflects the *p*_T_ distribution of the W, regardless of whether the lepton was produced in a top-quark decay or in a W+jets event. With suitable corrections, discussed below, the lepton *p*_T_ spectrum can therefore be used to predict the  spectrum for SM single-lepton backgrounds.

**Fig. 1:**
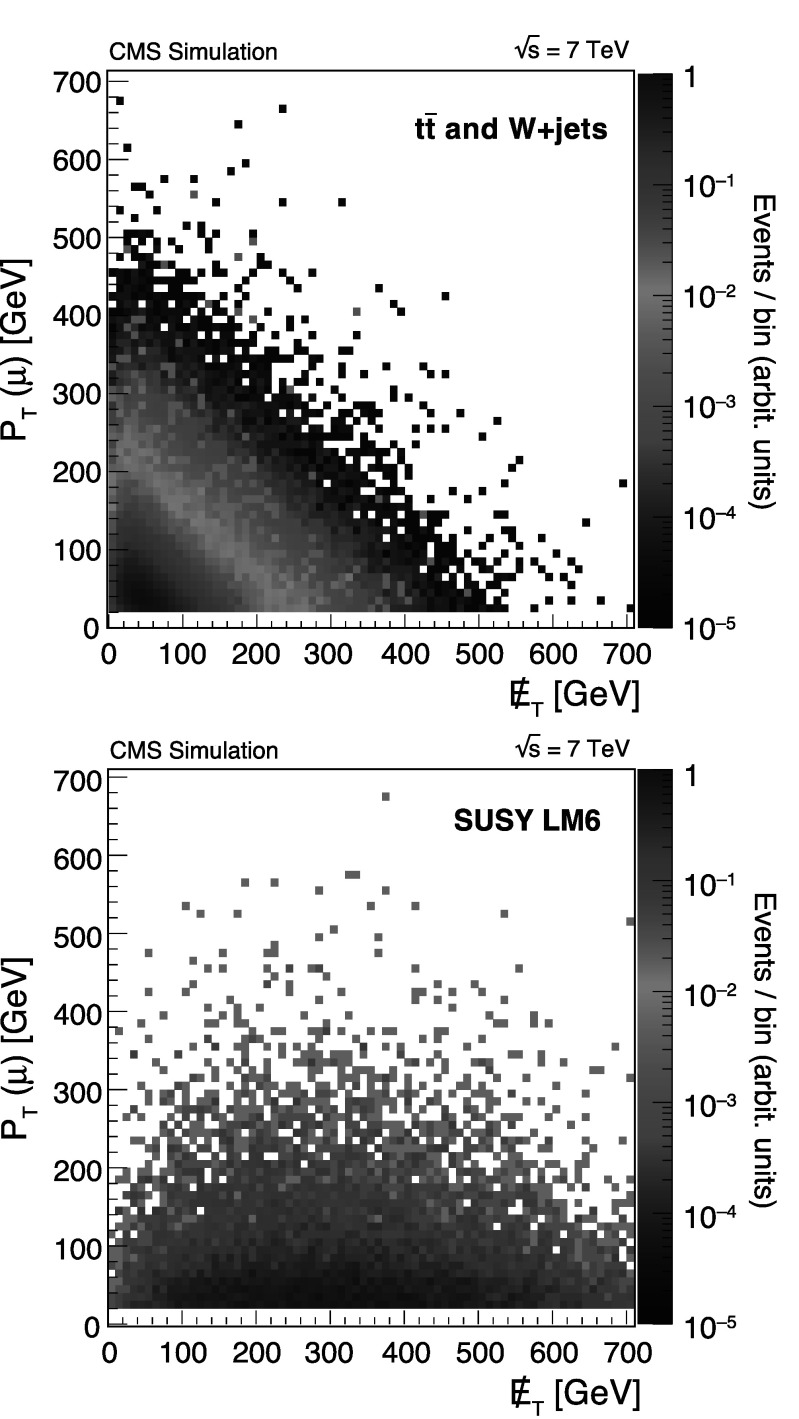
Distributions of muon *p*_T_ vs.  in the *μ* channel for simulated $\mathrm{t}\overline{\mathrm{t}}$ and W+jets events (*top*) and for the LM6 SUSY benchmark model (*bottom*)

The  distribution in many SUSY models is dominated by the presence of two LSPs. In contrast to the SM backgrounds, the  and lepton *p*_T_ distributions in SUSY processes are therefore nearly decoupled. The  distribution for such models extends to far higher values than the lepton spectrum. Figure 1 shows the relationship between the lepton-*p*_T_ and  distributions in the laboratory frame for two simulated event samples: (i) the predicted SM mixture of $\mathrm{t}\overline{\mathrm{t}}$ and W+jets events and (ii) the SUSY LM6 benchmark model. When taken from data, the upper-left region in Fig. 1 (top) provides the key control sample of high-*p*_T_ leptons from SM processes. This region typically has very little contamination from SUSY events, which populate the high- region but have relatively low lepton *p*_T_ values.

The lepton *p*_T_ spectrum is measured with a muon control sample defined by the preselection criteria and the *H*_T_ requirements. Unlike the signal region, no  requirement is applied, because even a modest one () would bias the high end of the lepton *p*_T_ spectrum, which is critical for making the background prediction. Only muon events are used as a control sample, because the QCD multijet background is significant in the low- region of the electron sample. The number of events that are common to both the control sample and the signal region is small. For example, the overlap as measured in simulated $\mathrm{t}\overline{\mathrm{t}}$ events is 3.6 % for *H*_T_≥750 GeV, , and *p*_T_≥250 GeV. Because no  requirement is placed on the muon control sample, a small amount of QCD background remains and must be measured and subtracted. The scaling from the muon to the electron samples is obtained by fitting their ratio in the data over the range , with systematic uncertainties evaluated by varying the fit range. The resulting correction factor is *N*(e)/*N*(*μ*)=0.88±0.03±0.03, where the uncertainties are statistical and systematic, respectively.

To use the lepton spectrum to predict the  spectrum in single-lepton SM background processes, three main issues must be understood: (i) the effect of the W-boson polarization in both $\mathrm{t}\overline{\mathrm{t}}$ and W+jets events, (ii) the effect of the applied lepton *p*_T_ threshold, and (iii) the difference between the experimental resolutions on the measurements of lepton *p*_T_ and .

The status of theoretical and experimental knowledge of W-boson polarization in $\mathrm{t}\overline{\mathrm{t}}$ and in W+jets events is discussed in Sect. 3. The helicity zero polarization state results in a forward–backward symmetric angular distribution of the lepton and the neutrino in the W rest frame (with respect to the W momentum direction), leading to identical lepton and neutrino spectra in the laboratory frame. In contrast, the helicity ±1 states result in angular asymmetries that lead to somewhat different lepton and neutrino *p*_T_ spectra in the laboratory frame. These effects are taken into account by applying correction factors obtained from simulation to the measured lepton spectrum, with uncertainties as described in Sect. 8.

The second key issue in the Lepton Spectrum method is the effect of the threshold (*p*_T_>20 GeV) applied to the leptons in both the signal and control samples. Because of the anticorrelation between the lepton *p*_T_ and the  arising from non-zero W-boson helicity states, the threshold requirement removes SM background events in the high- signal region but not the events in the control sample with high-*p*_T_ muons that are used to predict the high tail of the  spectrum. For the $\mathrm{t}\overline{\mathrm{t}}$ background, this effect partially compensates for the bias from the W polarization. For W+jets events, in contrast, the polarization effects for W^+^ and W^−^ approximately cancel, but the lepton *p*_T_ threshold shifts the predicted yield upward. Correction factors from simulation are used to account for these effects (as well as for polarization effects), which are well defined and understood.

Finally, the resolution on the reconstructed  is poorer than that for the lepton *p*_T_, so the  spectrum is somewhat broadened with respect to the prediction from the lepton spectrum. We measure  resolution functions in the data using QCD multijet events obtained with a set of single-jet triggers spanning the range from *E*_T_≥30 GeV to *E*_T_≥370 GeV. These resolution functions, or templates, quantify the  resolution as a function of the number of jets and the *H*_T_ of the event. These templates are used to smear the measured lepton momenta. Because the templates are taken from data, they include not only the intrinsic detector resolutions, but also acceptance effects. The overall effect of the smearing is modest, changing the background prediction by 5–15 %, depending on the  threshold applied.

The raw background predictions for the single-lepton background are corrected to account for the effects described above, as well as for the small contamination of the single-lepton control sample arising from dilepton and single-*τ* events with high-*p*_T_ leptons. These backgrounds are measured separately, as described below. The overall correction factor is defined such that the single-lepton prediction in a given signal region in simulation matches the yield from single-lepton processes.

The predicted single-lepton background yield varies from about 150 events for the signal region with  and *H*_T_≥500 GeV to about 3 events for the region with  and *H*_T_≥1000 GeV. These predictions, as well as the expectations from simulation, are presented in Tables 2, 3, and 4 and discussed in more detail in Sect. 5.4.

**Table 2:** Event yields for the Lepton Spectrum method for *H*_T_≥500 GeV. The upper part of the table gives the background predictions that are based on simulated (MC) event samples and the yield for the SUSY signal points LM3 and LM6. The lower part gives the backgrounds predicted using control samples in the data (data-driven prediction). The actual yield observed in data is given at the bottom, with the separate muon and electron yields given in parentheses (*N*_*μ*_,*N*_e_) after the total yield. The uncertainties on the background predictions are statistical and systematic. The MC yields are not used in setting limits and are included only for reference. The uncertainties on the MC yields are statistical only

range [GeV]	[250, 350)	[350, 450)	[450, 550)	≥550
MC yields				
1 *ℓ*	146.7±2.1	34.8±1.1	8.5±0.6	2.9±0.3
Dilepton	19.9±0.5	3.8±0.2	0.7±0.1	0.3±0.1
1 *τ*	30.6±0.9	7.9±0.5	2.1±0.3	0.8±0.2
Z+jets	1.3±0.8	<0.1	<0.1	<0.1
Total (MC)	198.6±2.5	46.5±1.2	11.3±0.6	4.0±0.4
SUSY LM3 (MC)	266.3±3.7	91.0±2.2	23.3±1.1	9.9±0.7
SUSY LM6 (MC)	23.4±0.3	20.0±0.3	13.4±0.2	10.8±0.2
Data-driven prediction				
1 *ℓ*	109±13±18	32.0±7.5±5.8	3.9±2.7±1.2	3.1±2.3±1.0
Dilepton	15.8±1.9±1.8	3.0±0.9±0.5	0.5±0.3±0.2	0.1±0.2±0.2
1 *τ*	33.0±1.8±1.7	8.9±1.0±0.5	2.1±0.5±0.2	1.1±0.3±0.2
QCD	0.0±1.0±1.2	0.0±1.0±1.2	0.0±1.0±1.2	0.0±1.0±1.2
Z+jets (MC)	1.3±0.8±1.3	<0.1	<0.1	<0.1
Total (predicted)	159±14±18	44.0±7.7±6.0	6.6±2.9±1.7	4.3±2.6±1.6
Data (observed)	163 (84,79)	46 (21,25)	9 (8,1)	2 (1,1)

**Table 3:** Event yields for the Lepton Spectrum method for *H*_T_≥750 GeV. Further details are given in the Table 2 caption

range [GeV]	[250, 350)	[350, 450)	[450, 550)	≥550
MC yield				
1 *ℓ*	47.3±1.2	14.9±0.7	5.4±0.4	2.7±0.3
Dilepton	8.2±0.4	2.3±0.2	0.6±0.1	0.3±0.1
1 *τ*	9.2±0.5	3.0±0.3	1.2±0.2	0.7±0.2
Z+jets	0.7±0.6	<0.1	<0.1	<0.1
Total (MC)	65.4±1.5	20.2±0.8	7.2±0.5	3.6±0.4
SUSY LM3 (MC)	114.6±2.5	47.1±1.6	16.1±0.9	8.6±0.7
SUSY LM6 (MC)	14.9±0.3	13.8±0.2	10.3±0.2	9.8±0.2
Data-driven prediction				
1 *ℓ*	41.7±8.7±5.4	11.7±5.0±1.9	2.6±2.3±0.6	3.1±2.4±0.8
Dilepton	5.9±1.1±0.7	1.3±0.5±0.2	0.5±0.2±0.1	0.1±0.1±0.3
1 *τ*	9.6±0.9±0.6	3.1±0.6±0.3	1.1±0.3±0.2	0.8±0.2±0.1
QCD	0.0±0.2±0.4	0.0±0.2±0.4	0.0±0.2±0.4	0.0±0.2±0.4
Z+jets (MC)	0.7±0.6±0.7	<0.1	<0.1	<0.1
Total (predicted)	57.9±8.9±5.6	16.2±5.0±2.0	4.2±2.4±0.8	4.0±2.4±1.0
Data (observed)	48 (27,21)	16 (7,9)	5 (4,1)	2 (1,1)

**Table 4:** Event yields for the Lepton Spectrum method for *H*_T_>1000 GeV. Further details are given in the Table 2 caption

range [GeV]	[250, 350)	[350, 450)	[450, 550)	≥550
MC yield				
1 *ℓ*	13.4±0.6	4.8±0.4	2.1±0.3	1.3±0.2
Dilepton	2.7±0.2	1.0±0.1	0.3±0.1	0.2±0.1
1 *τ*	2.1±0.2	0.7±0.1	0.5±0.1	0.4±0.1
Z+jets	0.5±0.5	<0.1	<0.1	<0.1
Total (MC)	18.8±0.9	6.4±0.5	2.9±0.3	1.9±0.2
SUSY LM3 (MC)	38.1±1.4	18.3±1.0	7.0±0.6	5.5±0.5
SUSY LM6 (MC)	7.0±0.2	6.0±0.2	4.6±0.1	5.2±0.2
Data-driven prediction				
1 *ℓ*	11.7±4.6±1.8	5.5±3.6±1.0	2.0±2.2±0.6	3.1±2.3±1.0
Dilepton	1.2±0.6±0.1	0.4±0.4±0.1	0.2±0.2±0.1	0.1±0.2±0.2
1 *τ*	3.0±0.5±0.5	0.9±0.3±0.2	0.4±0.2±0.2	0.8±0.2±0.2
QCD	0.0±0.1±0.1	0.0±0.1±0.1	0.0±0.1±0.1	0.0±0.1±0.1
Z+jets (MC)	0.5±0.5±0.5	<0.1	<0.1	<0.1
Total (predicted)	16.4±4.7±1.9	6.8±3.6±1.0	2.6±2.2±0.6	4.0±2.4±1.0
Data (observed)	14 (7,7)	4 (1,3)	0 (0,0)	2 (1,1)

### Estimation of non-single-lepton backgrounds

The non-single-lepton backgrounds include dilepton events in several categories, events with W→*τν* followed by *τ*→*ℓ* decays (in either $\mathrm{t}\overline{\mathrm{t}}$ or W+jets events), and QCD multijet processes. These subdominant backgrounds are estimated using control samples in data, in conjunction with information from simulation. The contribution from Drell-Yan and Z+jets is very small and is estimated directly from simulation.

Dilepton background events (including the *τ* as one of the leptons) contain at least two neutrinos, so these events can be important in the tails of the  distributions. These backgrounds are divided into the following categories: (i) 2*ℓ* events with one lost or ignored lepton (*ℓ*=e,*μ*), (ii) *ℓ*+*τ* events with *τ*→hadrons, and (iii) *ℓ*+*τ* events with *τ*→lepton. A lost lepton is one that is either not reconstructed or is out of the detector acceptance. An ignored lepton is one that is reconstructed but fails either the lepton-identification requirements (including isolation) or the *p*_T_ threshold requirement.

To estimate the background from dilepton events with lost or ignored leptons, we compute the ratio of the combined yield of dilepton events in the ee, e*μ*, and *μμ* channels in data to the corresponding combined yield in simulated event samples. This ratio, which is 0.91±0.07 for *H*_T_≥500 GeV, 0.93±0.15 for *H*_T_≥750 GeV, and 0.87±0.37 for *H*_T_≥1000 GeV, is used to rescale the  distribution of dilepton events that appear in the signal region in simulation. (Events within 20 GeV of the nominal Z mass are excluded in the e^+^e^−^ and *μ* ^+^ *μ* ^−^ channels.) This approach is used because the dilepton control sample in data is small, and using it to obtain the shapes of  distributions would result in large statistical uncertainties. For all  bins above 250 GeV, the predicted yield from this background contribution is less than 6 events, and for all  bins above 350 GeV, the yield is at or below 1 event. The  distribution associated with the reconstructed dilepton events in data is well described by the simulation.

Dilepton events can also involve *τ* decays, either *τ*→ hadrons or *τ*→*ℓ*. The  distributions in the dilepton events in data, when suitably modified to reflect the presence of a leptonic or hadronic *τ* decay, provide an accurate description of the shape of the  distribution of these backgrounds. Thus, to estimate the shape from the *τ*→ hadrons background, we effectively replace a lepton in a reconstructed dilepton event with a hadronic *τ* jet. Both hadronic and leptonic *τ* response functions are used, providing a probability distribution for a *τ* to produce a jet or a lepton with a given fraction *p*_T_(jet)/*p*_T_(*τ*) or *p*_T_(*ℓ*)/*p*_T_(*τ*). These response functions, obtained from simulation, are computed in bins of *p*_T_(*τ*). This procedure can change the total number of jets above threshold in the event, as well as other properties such as *H*_T_ and , which are recalculated. Simulated event samples are used to determine, for each of these processes *i*, the ratio $r_{i}=N_{\mathrm{feed}}^{i}/N_{\mathrm{control}}$ of the number of events observed in the single-lepton channel to the number of events in the control sample, as a function of . This procedure effectively normalizes all such contributions to the control samples in data. For all  bins above 250 GeV, the number of dilepton events with a *τ*→hadrons decay is predicted to be about 7 events or less and is much smaller in the higher  bins. The number of dilepton events with a *τ*→*ℓ* decay is predicted to be less than 3 events for all  bins above 250 GeV and is much smaller in the higher  bins.

Estimates for the *τ*→*ℓ* single-lepton backgrounds from $\mathrm{t}\overline{\mathrm{t}}$ and W+jets processes are based on a procedure similar to that used for the dilepton backgrounds, but in this case the single-lepton sample itself is used as the control sample. The  distribution obtained by applying the *τ*→*ℓ* response function to the data is rescaled by a ratio from simulation that gives the yield of *τ*→*ℓ* background events divided by the yield of events in the single-lepton control sample, as a function of . The number of background events from the single *τ*→*ℓ* contribution falls from 33 for *H*_T_≥500 GeV and  to 1.1 event for *H*_T_≥500 GeV and .

**Fig. 2:**
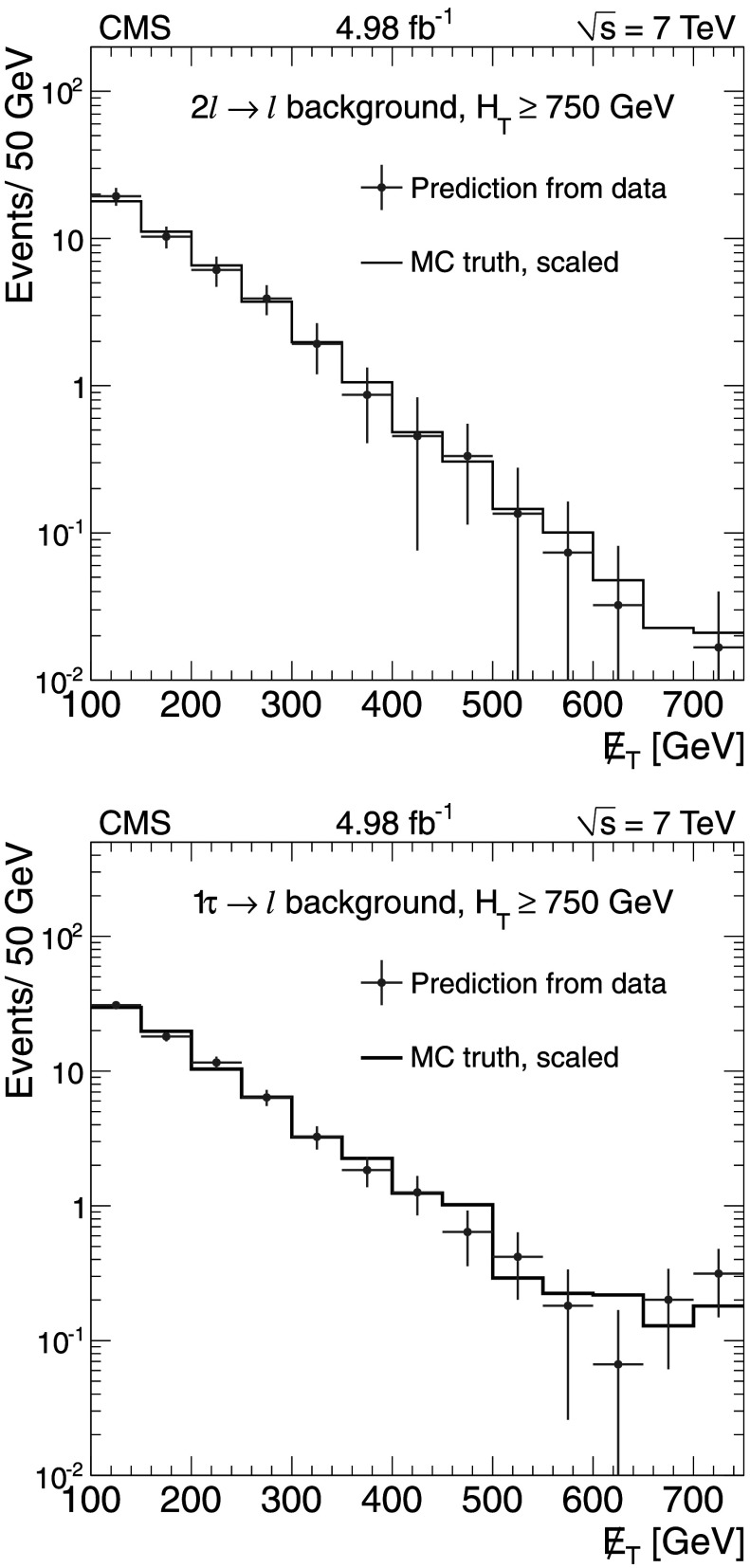
Predictions for dilepton and *τ*→*ℓ* backgrounds after requiring *H*_T_≥750 GeV: control samples in data (*green points with error bars*) vs. MC predictions (*black solid histogram*) for (*top*) dilepton background and (*bottom*) *τ*→*ℓ* background. The MC prediction has been scaled to the integral of the data prediction (Color figure online)

The background predictions in data are shown in Fig. 2, where the expectation based on simulation is shown for comparison. The total predicted dilepton plus single *τ*→*ℓ* background yield ranges from about 50 events for *H*_T_≥500 GeV and  to about 1 event for *H*_T_≥1 TeV and . All of these predictions, as well as the expectations from simulation, are presented in Tables 2, 3, and 4, which are discussed in more detail in Sect. 5.4.

Background from QCD multijet events is suppressed to a level well below 1 event in nearly all signal regions, as shown in Tables 2, 3, and 4. The QCD multijet background is determined by first defining a control sample with small missing transverse momentum () and with a lepton impact parameter relative to the beam spot |*d*_0_|>0.02 cm. These requirements select a sample with little contamination from other SM processes such as $\mathrm{t}\overline{\mathrm{t}}$ and W+jets processes. Using this control sample, we measure the shape of the distribution in the combined relative isolation variable, $I_{\mathrm{rel}}^{\mathrm{comb}}$ (see Sect. 4). The shape of this distribution has very little correlation to  or to the lepton impact parameter (*d*_0_), and so can be applied in the high- signal regions. For each signal region in the data, we determine the background at low values of $I_{\mathrm{rel}}^{\mathrm{comb}}$ by first scaling the measured QCD multijet background shape in the relative isolation variable to the high-$I_{\mathrm{rel}}^{\mathrm{comb}}$ sideband of the signal region. The shape is then used to extrapolate the yield to the low-$I_{\mathrm{rel}}^{\mathrm{comb}}$ signal region. In the high- signal regions, some non-QCD SM background can be present at high $I_{\mathrm{rel}}^{\mathrm{comb}}$, where the QCD background shape is normalized. We therefore subtract the estimated background from $\mathrm{t}\overline{\mathrm{t}}$, W+jets, and Z+jets from this region. These yields are taken from simulation, with systematic uncertainties determined from a comparison with a control region in the data.

### Results from the Lepton Spectrum method

Tables 2, 3, and 4 compare the background yields predicted from the control samples in data with the yields obtained directly from simulation for *H*_T_≥500 GeV, *H*_T_≥750 GeV, and *H*_T_≥1000 GeV, respectively. We observe that the single-lepton background is the dominant contribution in all regions. The various sources of uncertainties associated with these background determinations are discussed in Sect. 9. Finally, the yields observed in the signal regions in the data, which are listed at the bottom of each table, are consistent with the total background predictions based on the control samples. Thus, we observe no evidence for any excess of events in the data above the SM contributions.

**Fig. 3:**
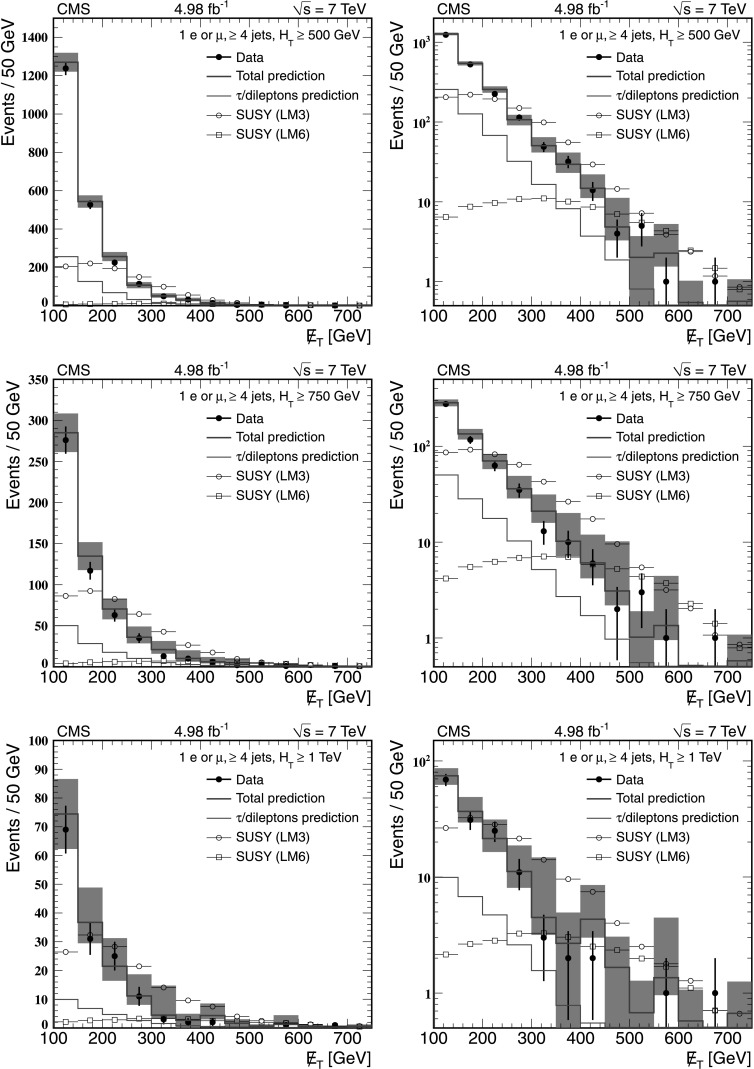
Lepton Spectrum method: observed  distributions in data (*filled points with error bars*) compared with predicted  distributions (*green bars*) in the combined electron and muon channels, on linear (*left*) and logarithmic (*right*) scales. Three different *H*_T_ thresholds are applied: *H*_T_≥500 GeV (*upper row*), *H*_T_≥750 GeV (*middle row*), and *H*_T_≥1000 GeV (*lower row*) (Color figure online)

Figure 3 shows the  distributions in data for the combined muon and electron channels, with all of the selection requirements, except that on  itself. The distributions are shown for *H*_T_≥500 GeV, *H*_T_≥750 GeV, and *H*_T_≥1000 GeV, on both linear and logarithmic scales. The predicted  distribution (green-bar histogram) is a sum over three sources: single-lepton backgrounds (from $\mathrm{t}\overline{\mathrm{t}}$, single-top, and W+jets events), dilepton background from $\mathrm{t}\overline{\mathrm{t}}$, and single-*τ* events (from both $\mathrm{t}\overline{\mathrm{t}}$ and W+jets processes). The vertical span of the green bar corresponds to the statistical uncertainty on the background prediction. (The systematic uncertainties are computed in wider bins used for setting the limits and are given in Tables 2, 3, and 4.) In each signal region, the blue histogram shows the contribution from the dilepton and single-*τ* backgrounds only. It is evident that the single-lepton background is dominant in all cases. The  distributions for the SUSY benchmark models LM3 and LM6 are overlaid (not summed) for comparison. Systematic uncertainties and the interpretation are presented in Sect. 9.

## Lepton Projection method

### Overview of the Lepton Projection method

The Lepton-Projection (LP) method uses the difference between SM and SUSY processes in the correlation of the lepton transverse momentum and the missing transverse momentum. As previously discussed, in the SM processes the  corresponds to the neutrino in the decay of the W boson, either in W+jets or in $\mathrm{t}\overline{\mathrm{t}}$ events. The kinematics of W decays are dictated by the V–A nature of the W coupling to leptons and the helicity of the W boson, as discussed in Sect. 3. Since W bosons that are produced with high transverse momentum in W+jets events exhibit a sizable left-handed polarization, there is a significant asymmetry in the *p*_T_ spectra of the neutrino and charged lepton. A smaller asymmetry is expected in W bosons from t quark ($\bar{\mathrm{t}}$ antiquark) decays, which yield W bosons which are predominantly longitudinally polarized with smaller left-handed (right-handed) components for W^+^ (W^−^).

We have measured the fraction of the helicity states of the W boson using an angular analysis of leptonic W decays [21]. Since the total momentum of the W boson in these decays, and therefore its center-of-mass frame, cannot be accurately determined because the momentum of the neutrino along the beam axis cannot be measured, an observable that depends only on transverse quantities is used. A variable that is highly correlated with the cosine of the polar angle (with respect to the W boson flight direction) in the center-of-mass frame of the W boson is the “lepton projection variable”:

3 $$ L_{\mathrm{P}}= \frac{\boldsymbol{p}_{\mathrm{T}}(\ell) \cdot\boldsymbol{p}_{\mathrm{T}}({\mathrm{W}})}{|\boldsymbol{p}_{\mathrm{T}} ({\mathrm{W}})|^2}, $$

where ***p***_T_(*ℓ*) is the transverse momentum of the charged lepton and ***p***_T_(W) is the transverse momentum of the W boson. The latter quantity is obtained from the vector sum of the charged lepton transverse momentum and the missing transverse momentum in the event.

**Fig. 4:**
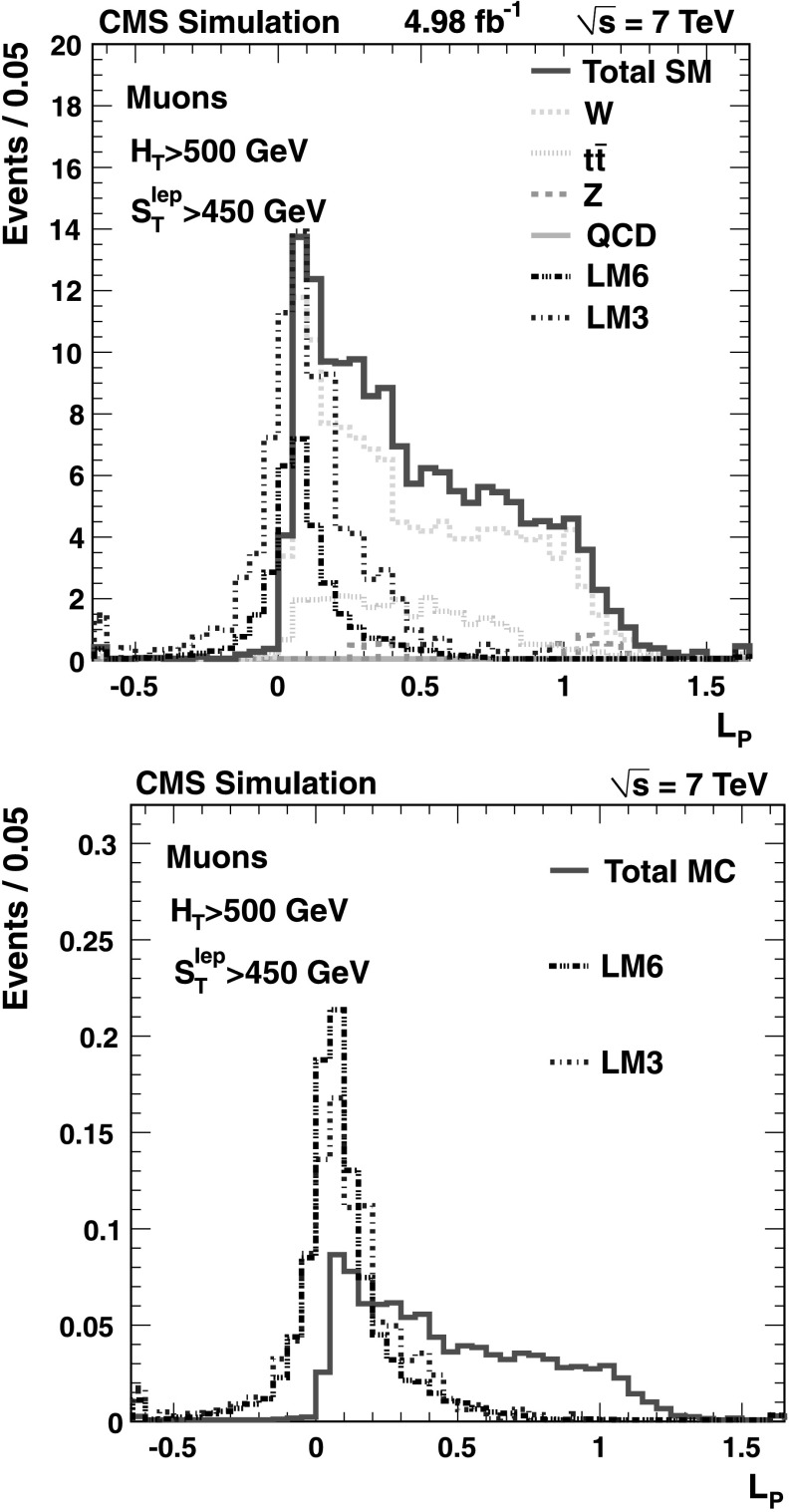
Distribution of *L*_P_ in SUSY and standard model processes from simulation. *Top*: all distributions are normalized to the integrated luminosity. The different contributions from SM processes are shown, whereas for SUSY two benchmark points, LM3 and LM6, are displayed. *Bottom*: the same distributions normalized to unity. The SM distribution is the sum of all the individual SM processes shown in the *top pane*. The quantity  is discussed in the text

Since SUSY decay chains result in large values of , and often result in relatively low values of the lepton momentum as well, the *L*_P_ distribution for SUSY events tends to peak near zero, whereas W+jets and $\mathrm{t}\overline{\mathrm{t}}$ yield a broad range of *L*_P_ values. This behavior is illustrated in Fig. 4, which compares the *L*_P_ distribution from both SM processes and from two representative SUSY benchmark points (LM3 and LM6).

In the *L*_P_ method, two regions in *L*_P_ are defined: the region with *L*_P_<0.15 is used as the signal region; the region with *L*_P_>0.3 is used as the control region, i.e., a sample that is depleted in the signal expected and is instead dominated by SM processes. These regions are selected using simulated event samples of W+jets, Z+jets, and $\mathrm{t}\overline{\mathrm{t}}$, that are collectively referred to as electroweak (EWK) processes in what follows, as well as with simulated SUSY events with SUSY particle masses near the region currently under exploration.

### Background estimation in the *L*_P_ method

The key ingredient of the analysis is the estimate of the number of events in the signal region from the SM processes. We define a translation factor,

4 $$ R_\mathrm{CS} = \frac{N_\mathrm{MC}(L_{\mathrm{P}}<0.15)}{N_\mathrm {MC}(L_{\mathrm{P}}>0.3)}, $$

which is the ratio of the number of events in the signal and control regions for the EWK processes. The translation factor is obtained from MC simulation of the EWK processes, and the uncertainties on this factor are included in the systematic uncertainty of the background estimate. In the case of muons, where the background from QCD multijets is negligible, the total number of events predicted from SM processes in the signal region, $N_{\mathrm{SM}}^{\mathrm{pred}}(L_{\mathrm{P}}<0.15)$, can be determined directly from the number of events observed in the data in the control region, *N*_data_(*L*_P_>0.3):

5 $$ N_\mathrm{SM}^\mathrm{pred}(L_{\mathrm{P}}<0.15) = R_\mathrm{CS} \cdot N_\mathrm{data}(L_{\mathrm{P}}>0.3). $$

In the case of the electrons, the presence of events from QCD multijet processes necessitates an independent evaluation of this background prior to the application of the translation factor for EWK processes.

The number of events estimated with this method is then compared to the number of events observed in the data in the signal region, *N*_data_(*L*_P_<0.15), for indications of an excess of events over the SM expectation. The analysis is performed in different regions of the event mass scale. To characterize the latter without affecting the correlation of the charged lepton and the neutrino in SM events, the scalar sum of the lepton transverse momentum and the missing transverse momentum, $S_{\mathrm{T}}^{\mathrm{lep}}$, is used: . For W decays, $S_{\mathrm{T}}^{\mathrm{lep}}\approx p_{\mathrm{T}}({\mathrm{W}})$ at large values of *p*_T_(W).

In order to make the search optimization less dependent on the unknown energy scale of a new physics signal, the analysis is performed in disjoint ranges of $S_{\mathrm{T}}^{\mathrm{lep}}$ and the results in these ranges are combined. In addition, the selection is also binned in a second dimension, the *H*_T_ variable, defined in Eq. (2).

As indicated in Table 1, the event selection used in this analysis is slightly different from the corresponding one in the LS analysis. To increase the sensitivity to SUSY decays, this analysis requires three or more jets. While this results in a significant increase in W+jets events, the additional SM background is mostly concentrated in the control region in *L*_P_.

The event yields in the muon and electron channels, as predicted from simulation, are shown in Table 5. As discussed previously, the dominant backgrounds to the lepton plus jets and  signature arise from the production and decay of W+jets and $\mathrm{t}\overline{\mathrm{t}}$. The production of single W bosons in association with jets, and with large transverse momenta, is in general the larger of the two, especially at lower jet multiplicities. The majority of the $\mathrm{t}\overline{\mathrm{t}}$ background arises from semi-leptonic $\mathrm{t}\overline{\mathrm{t}}$ decays, with fully leptonic $\mathrm{t}\overline{\mathrm{t}}$ decays in which a lepton is either ignored or not reconstructed contributing about 20 % of the total $\mathrm{t}\overline{\mathrm{t}}$ background.

**Table 5:** Expected event yields in the signal region (*L*_P_<0.15) from simulation. These yields are for *H*_T_>500 GeV. These MC values are only listed for illustration purposes

*L*_P_<0.15	Muons: $S_{\mathrm{T}}^{\mathrm{lep}}$ range [GeV]			Electrons: $S_{\mathrm{T}}^{\mathrm{lep}}$ range [GeV]		
	[250–350]	[350–450]	[450–∞]	[250–350]	[350–450]	[450–∞]
$\mathrm{t}\overline{\mathrm{t}}$ (*ℓ*)	50.0±1.0	15.3±0.5	4.8±0.3	37.9±0.8	11.0±0.4	3.6±0.2
$\mathrm{t}\overline{\mathrm{t}}$ (*ℓℓ*)	12.4±0.4	3.9±0.2	1.2±0.1	10.4±0.4	2.9±0.2	0.8±0.1
W	66.2±2.0	35.6±1.4	26.0±1.2	48.9±1.7	24.2±1.2	20.9±1.1
Z	2.1±1.0	0.4±0.4	0.0±0.2	1.4±0.8	0.0±0.2	0.0±0.2
Total MC	130.8±2.4	55.3±1.6	32.0±1.3	98.6±2.1	38.1±1.3	25.3±1.1
LM3	136.8±3.8	89.1±3.1	53.9±2.4	111.7±3.4	70.8±2.7	47.0±2.2
LM6	8.4±0.2	11.0±0.2	24.9±0.3	6.7±0.2	8.5±0.2	20.5±0.3

A source of background, which is not listed in Table 5, stems from QCD multijet events in which a jet is misreconstructed as a lepton. The simulation indicates that the magnitude of this background is small in the control region and negligible in the signal region. Nevertheless, since the uncertainties in simulating these backgrounds can be significant, we use control data samples to estimate the background in the muon and electron channels.

To estimate the background from QCD multijets in the muon final state, we use the relative combined isolation, $I_{\mathrm{comb}}^{\mathrm{rel}}$, of the muon. Multijet events are expected to populate the region at high values of $I_{\mathrm{comb}}^{\mathrm{rel}}$, whereas muons from SUSY decays are isolated and thus have low values of $I_{\mathrm{comb}}^{\mathrm{rel}}$. We employ an additional control data sample, which is specially selected to be enriched in QCD multijets, to determine the ratio of multijet events at low values of the relative isolation. Using this ratio and the number of multijet events expected in the control region of the sample passing the preselection requirements, we estimate the background from multijet events in the signal region to be always smaller than 1 % of the EWK backgrounds. This level of background is negligible and is thus ignored in what follows.

The main sources of electrons in QCD multijet events are misidentified jets and photon conversions. This background is expected to be more substantial than the corresponding one in the muon sample, and its estimate exhibits a large dependence on the details of the simulation. For this reason, we estimate this background from control samples in data. The method relies on the inversion of one or more of the electron identification requirements in order to obtain a sample of anti-selected events, which is dominated by jets misidentified as electrons. We find that the inversion of the requirements on the spatial matching of the calorimeter cluster and the charged-particle track in pseudorapidity and azimuth leaves the relative fraction of the different background sources in QCD multijets unchanged. Moreover, to increase the number of events in this control sample, the requirements on *d*_0_ and *d*_*z*_ are removed, while the isolation requirement is loosened. These changes to the event selection have a negligible effect on the *L*_P_ distribution in the data. In the simulated event samples, it is found that the *L*_P_ distribution from the control sample events provides a good description of the corresponding distribution from QCD background passing all selection requirements.

The *L*_P_ distribution obtained with this control sample is used as a template to fit, along with the *L*_P_ distribution from EWK processes, the *L*_P_ distribution in the data. In this fit, the EWK template is taken from simulation. This approach, which provides a template obtained from data for the QCD contamination, was applied in the measurement of the polarization of high-*p*_T_ W bosons [21]. The fit is performed in the control region (*L*_P_>0.3), where the possible presence of signal is highly suppressed. The numbers of QCD and EWK events obtained by the fit are used to estimate the total SM contamination in the signal region (*L*_P_<0.15). The method for estimating the number of SM events expected in the signal region is applied in each range of $S_{\mathrm{T}}^{\mathrm{lep}}$ and *H*_T_.

**Fig. 5:**
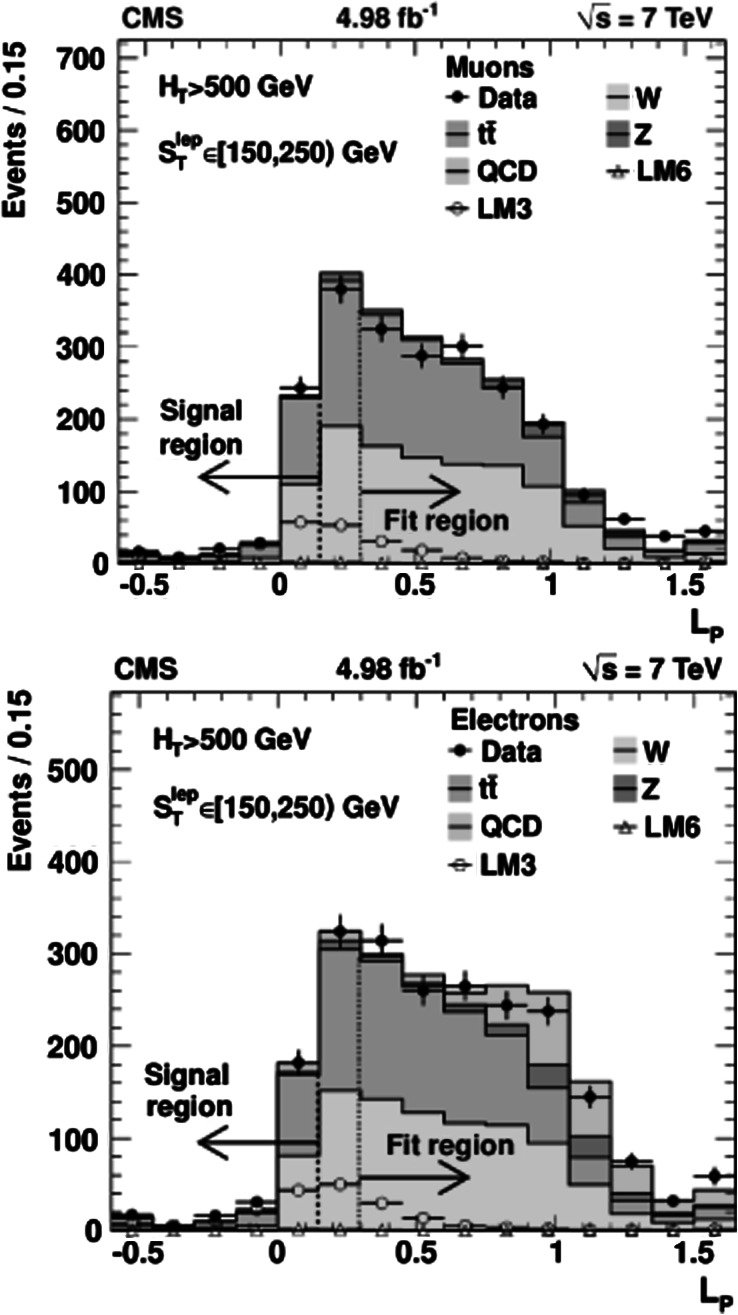
Fit results on data for $150 < S_{\mathrm{T}}^{\mathrm{lep}}<250~\mathrm{GeV}$, in the muon (*top*) and electron (*bottom*) search samples. The fit is performed in the control region (*L*_P_>0.3) and the result is extrapolated into the signal region (*L*_P_<0.15)

The method for estimating the SM expectation in the signal region is checked using two different control samples, where both the fit and signal regions have a negligible expected SUSY yield. The first sample is defined as all events satisfying the preselection requirements but confined to low values of $S_{\mathrm{T}}^{\mathrm{lep}}$: $150<S_{\mathrm{T}}^{\mathrm{lep}}<250~\mathrm{GeV}$. The method described above is employed to predict the number of events expected in the signal region for both muons and electrons. This prediction is found to be fully consistent with the number of events observed in the data in the signal region. The results of the fits and the yields of QCD and EWK events in the region of low $S_{\mathrm{T}}^{\mathrm{lep}}$ (<250 GeV) are displayed in Fig. 5 for the electron and muon samples. As can be seen in Fig. 5, the QCD contamination in the signal region, *L*_P_<0.15, is negligible, as expected, since low values of *L*_P_ favor events with low-*p*_T_ leptons and high . The second sample, used only for events with muons, is collected with a separate trigger without any requirements on *H*_T_ or . The muon transverse momentum threshold is raised to *p*_T_(*μ*)>35 GeV, while the *H*_T_ threshold is lowered from 500 GeV to 200 GeV and the jet multiplicity requirement is reversed, to be fewer than three jets. Given these requirements on *H*_T_ and on the jet multiplicity, this control sample is dominated by SM processes. It is found that the estimated background agrees well with the number of events seen in the signal region *L*_P_<0.15.

### Results of the *L*_P_ method

**Fig. 6:**
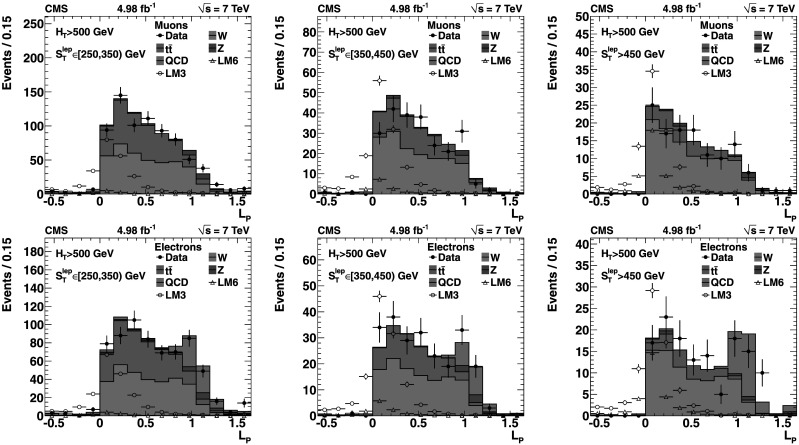
Data and fit results for the predictions for the *L*_P_ distribution, for events in the search sample, in different $S_{\mathrm{T}}^{\mathrm{lep}}$ regions. *Top plots* for the muon channel; *bottom plots* for the electron channel. *Left*: $250<S_{\mathrm{T}}^{\mathrm{lep}}<350~\mathrm{GeV}$, *center*: $350<S_{\mathrm{T}}^{\mathrm{lep}}<450~\mathrm{GeV}$, and *right*: $S_{\mathrm{T}}^{\mathrm{lep}}>450~\mathrm{GeV}$

**Fig. 7:**
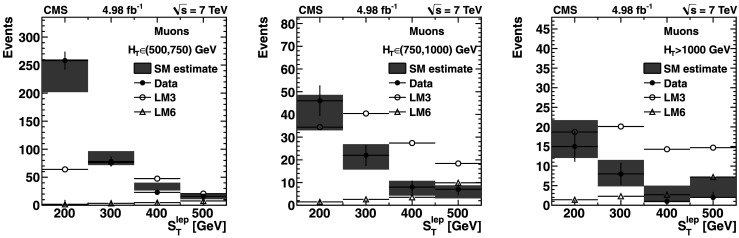
Comparison of the number of events observed in the data and the expectations from the background estimation methods for the electron channel, in the different $S_{\mathrm{T}}^{\mathrm{lep}}$ bins. *Left*: 500<*H*_T_<750 GeV; *Center*: 750<*H*_T_<1000 GeV; *Right*: *H*_T_>1000 GeV. The *error bars* indicate the statistical uncertainty of the data only, while the *green band* indicates the total statistical and systematic uncertainty on the background estimate (Color figure online)

**Fig. 8:**
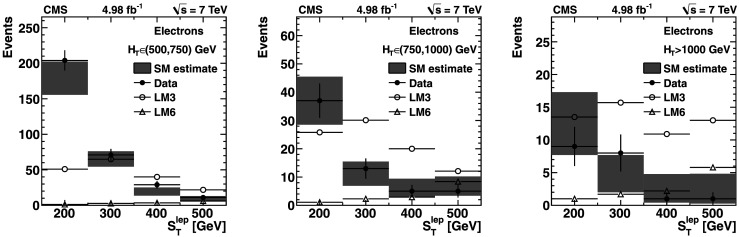
Comparison of the number of events observed in the data and the expectations from the background estimation methods for the muon channel, in the different $S_{\mathrm{T}}^{\mathrm{lep}}$ bins. *Left*: 500<*H*_T_<750 GeV; *Center*: 750<*H*_T_<1000 GeV; *Right*: *H*_T_>1000 GeV. The *error bars* indicate the statistical uncertainty of the data only, while the *green band* indicates the total statistical and systematic uncertainty on the background estimate (Color figure online)

The *L*_P_ distributions in three ranges of $S_{\mathrm{T}}^{\mathrm{lep}}$, are displayed in Fig. 6 for muons (top) and electrons (bottom). Tables 6 and 7 list the numbers of events observed and the number of events expected from all SM processes as presented above, in the signal region, for the muon and electron channels, respectively. The predictions, along with the numbers of events observed in each range of $S_{\mathrm{T}}^{\mathrm{lep}}$ and *H*_T_, are also displayed graphically in Fig. 8 for muons and in Fig. 7 for electrons. The uncertainties quoted in Table 7 correspond to the statistical uncertainty of the fit, while the predictions displayed in Fig. 7 include the total statistical and systematic uncertainty.

**Table 6:** Event yields in data and MC simulation for the muon sample. The results in the columns labeled “Total MC” are listed for reference only. The corresponding uncertainties statistical only

$S_{\mathrm{T}}^{\mathrm{lep}}$ range [GeV]	Total MC	Data	Total MC	SM estimate	Data
	Control region (*L*_P_>0.3)		Signal region (*L*_P_<0.15)		
500<*H*_T_<750 GeV					
[150–250)	1465±11	1297	261±3.2	261±7±24	258
[250–350)	452±5.2	383	99.3±2.1	84.1±4.2±7.3	78
[350–450)	154±3.1	128	40.2±1.4	33.3±3.0±2.6	23
≥450	59.2±1.8	50	18.6±1.0	15.7±2.2±2.0	16
750<*H*_T_<1000 GeV					
[150–250)	280±4.1	218	52.4±1.6	40.8±2.9±3.5	46
[250–350)	91.9±2.1	88	22.3±0.9	21.3±2.3±2.2	22
[350–450)	34.6±1.3	25	10.3±0.6	7.5±1.5±1.0	8
≥450	26.7±1.4	18	8.8±0.6	5.9±1.4±0.7	7
1000 GeV<*H*_T_					
[150–250)	92.3±2.5	76	20.5±1.0	16.9±1.9±1.7	15
[250–350)	32.9±1.3	31	8.7±0.8	8.2±1.5±1.0	8
[350–450)	10.9±0.7	7	4.6±0.4	2.9±1.1±0.6	1
≥450	11.9±0.8	12	4.6±0.5	4.6±1.4±0.7	2

**Table 7:** Event yields in data and predictions of the numbers of EWK and QCD events for the electron sample in bins of *H*_T_. The sum of predicted EWK events and predicted QCD events in the control region is constrained to be equal to the total number of data events. The background estimate used in comparing to the yields in the data is the result of the procedure described earlier and is listed in the row labeled “SM estimate”. The uncertainties for the QCD and EWK background estimates are statistical only. The uncertainties shown for the SM estimate are first the statistical uncertainty from the control region fit and second all other systematic uncertainties

$S_{\mathrm{T}}^{\mathrm{lep}}$ range [GeV]	QCD	EWK	Data	QCD	EWK	SM estimate	Data
	Control region (*L*_P_>0.3)			Signal region (*L*_P_<0.15)			
500<*H*_T_<750 GeV							
[150–250)	184±33	1122±45	1306	9.1±1.6	170±7	179±7±18	204
[250–350)	66±15	334±22	400	2.1±0.5	63.3±4.1	65.3±4.3±5.9	71
[350–450)	26.6±7.6	93±11	120	0.3±0.1	19.2±2.3	19.4±2.4±2.9	29
≥450	17.1±5.1	33.9±6.6	51	0.2±0.0	9.0±1.8	9.2±1.9±1.7	11
750<*H*_T_<1000 GeV							
[150–250)	39±15	210±20	249	1.9±0.7	35.1±3.3	37.0±3.5±4.8	37
[250–350)	5.8±5.5	59.2±9.1	65	0.2±0.2	11.0±1.7	11.2±2.0±1.8	13
[350–450)	<0.1	26.0±5.1	26	<0.1	6.3±1.2	6.3±1.2±1.5	5
≥450	8.7±3.4	22.3±5.0	31	0.1±0.0	6.7±1.5	6.8±1.6±1.5	5
1000 GeV<*H*_T_							
[150–250)	14.9±7.7	62±10	77	0.7±0.4	11.7±1.9	12.5±2.2±2.4	9
[250–350)	10.4±4.3	20.6±5.4	31	0.3±0.1	4.5±1.2	4.8±1.5±1.1	8
[350–450)	0.5±1.7	11.5±3.7	12	<0.1	2.6±0.8	2.6±1.2±0.9	1
≥450	4.4±2.5	6.6±2.9	11	0.0±0.0	2.5±1.1	2.6±1.3±0.9	1

All estimates of the total contribution expected from SM processes in the various bins in ($S_{\mathrm{T}}^{\mathrm{lep}},H_{\mathrm{T}}$) are consistent with the numbers of events observed in the data, with no visible excess from a potential SUSY signal. The result is interpreted as a limit in SUSY parameter space in the context of the CMSSM in Sect. 9.

## The Artificial Neural Network method

### Overview of the method

The Artificial Neural Network (ANN) method uses a multi-variate analysis to combine several event characteristics, other than , into a single variable that distinguishes signal from background. Signal events then preferentially populate a signal region in the two-dimensional plane of the ANN output (*z*_ANN_) and , and the sidebands in this plane provide an estimate of the residual background.

Four input variables drive the ANN. The first two are *n*_jets_, the number of jets with *p*_T_>40 GeV, and *H*_T_, the scalar sum of the *p*_T_ of each jet with *p*_T_>40 GeV. The SUSY signal typically has heavy particles decaying via complex cascades, and as such, is likely to produce more jets and larger *H*_T_ than SM backgrounds. The third variable is Δ*ϕ*(j_1_,j_2_), the angle between the two leading *p*_T_ jets in the transverse plane, which makes use of the greater likelihood that the two highest *p*_T_ jets are produced back-to-back in SM than in SUSY events. The final variable is *M*_T_, the transverse mass of the lepton and  system. In $\mathrm{t}\overline{\mathrm{t}}$ and W+jets events, the lepton and  generally arise from the decay of a W boson, and as a result, *M*_T_ peaks near the W boson mass, with larger values arising only when there are additional neutrinos from *τ* or semileptonic decays. By contrast, in SUSY events, *M*_T_ tends to be greater than the W mass because of  due to undetected LSPs.

**Fig. 9:**
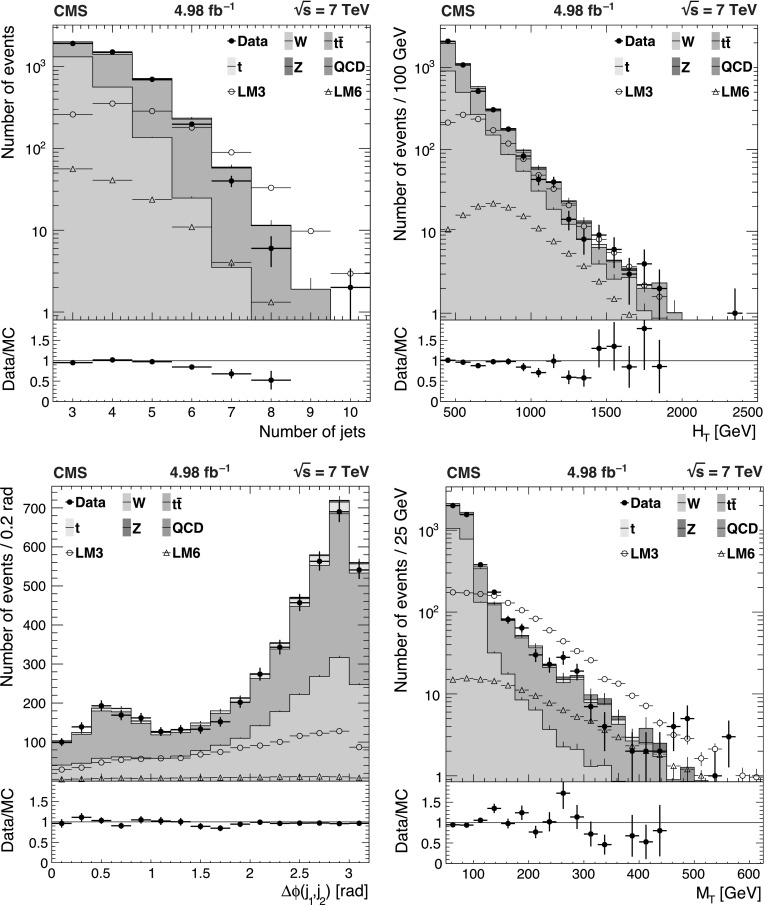
The distributions of *n*_jets_, *H*_T_, Δ*ϕ*, and *M*_T_ for data (*solid circles*), simulated SM (*stacked shaded histograms*), LM3 (*open circles*), and LM6 (*open triangles*) events after preselection. The *small plot beneath each distribution* shows the ratio of data to simulated SM yields. The muon and electron channels are combined

Figure 9 shows the distributions of these variables for simulated SM and SUSY events. The most powerful input variable is *M*_T_; *n*_jets_ and *H*_T_ also have considerable discriminating power. The Δ*ϕ*(j_1_,j_2_) variable is weaker, but it still improves the sensitivity of the search. Lepton *p*_T_ also discriminates between the SM and SUSY, but it is not included in the ANN because its strong correlation with  in the SM would spoil the background estimate. Additional variables either do little to improve sensitivity or introduce a correlation between *z*_ANN_ and . The input variables have similar distributions in the muon and electron channels, so we choose to train the ANN on the two channels combined, and use the same ANN for both. In general, the SM simulation describes the data adequately apart from a possible small structure near 130 GeV in the *M*_T_ distribution. Reweighting the simulation to match the *M*_T_ distribution in data does not affect the results of the analysis.

The ANN infrastructure uses standard Root utilities [42]. During training, weights are determined that minimize the root-mean-square deviation of background events from zero and signal events from unity. For the SUSY parameter space under study, our sensitivity depends only mildly on the details of the signal sample that trains the ANN. Specifically, for LM points 0 through 13 [30], the sensitivity is comparable (less than 30 % variation) whether the ANN is trained on LM0, LM6 or LM9, even though these three training samples have rather different characteristics. We select LM0 for training because it gives the best overall performance. The SM simulation provides the background sample.

**Fig. 10:**
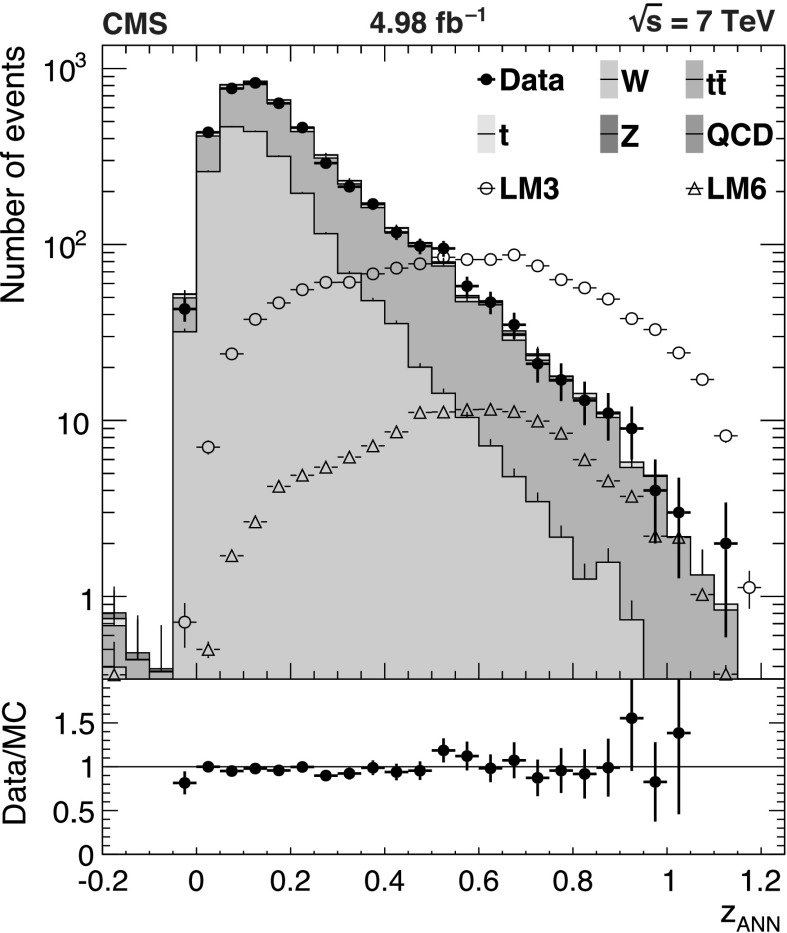
The *z*_ANN_ distribution of the data (*solid circles*) and simulated SM (*stacked shaded histograms*), LM3 (*open circles*), and LM6 (*open triangles*) events, after preselection. The *small plot beneath* shows the ratio of data to simulated SM yields

Figure 10 compares the distributions of *z*_ANN_ for data and SM simulation for all events surviving the preselection. The two distributions are consistent within the uncertainties. The SM contribution is concentrated at small values of *z*_ANN_, while the LM3 and LM6 SUSY distributions, which are also shown, extend to high values of *z*_ANN_ where the SM is suppressed.

We define two signal regions in the two-dimensional  and *z*_ANN_ plane. One region, referred to as the “low-” signal region, has *z*_ANN_>0.4 and , while the other, the “high-” signal region, has the same *z*_ANN_ range, but . The high- signal region minimizes the probability that the expected background fluctuates up to a LM6 signal when signal contamination is taken into account. We observe 10 events in the low- signal region and 1 event in the high- signal region.

### Background estimation using the ANN sidebands

**Fig. 11:**
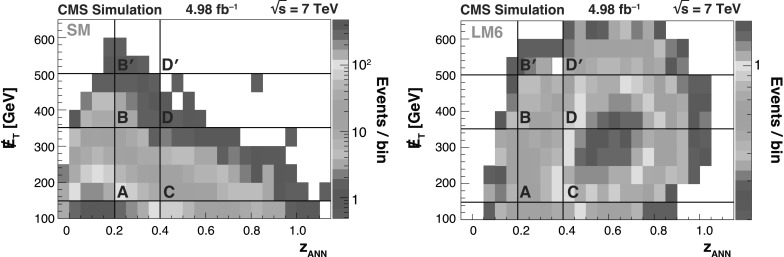
The yields of simulated SM (*left*) and LM6 (*right*) events in the  versus *z*_ANN_ plane. The regions D and D′ are the low- and high- signal regions. The sideband regions are also indicated

The sidebands in the two dimensional plane of  and *z*_ANN_ provide a strategy for estimating the background. The signal and sideband regions are shown in Fig. 11 and are denoted A, B, C, and D for the low- signal region and A, B′, C, and D′ for the high- signal region. The choice of boundaries for the sideband regions balances the competing needs of statistics and insensitivity to signal contamination against preserving similar event compositions in the signal and sideband regions.

**Fig. 12:**
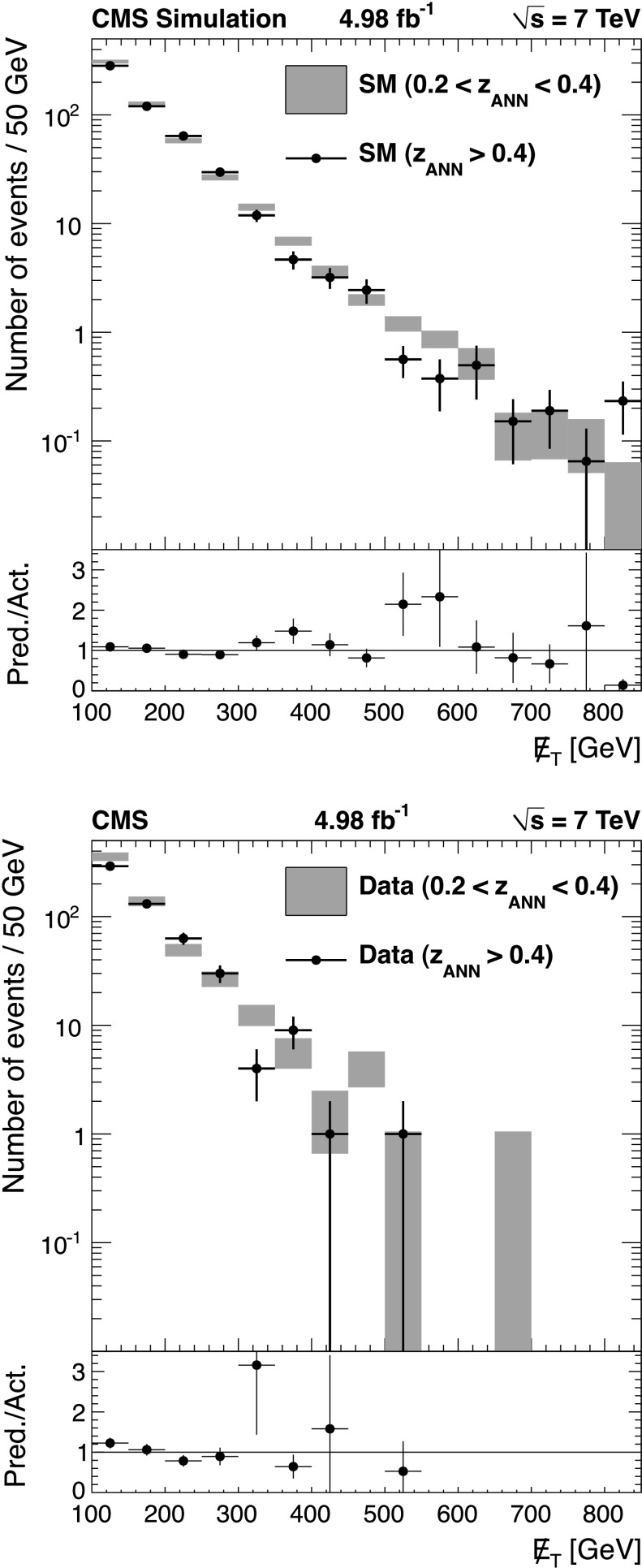
The  distributions of events in the *z*_ANN_ signal region (*solid circles*) and sideband (*green bars*) for simulated SM (*top*) and data (*bottom*) events. The distributions are normalized in the  sideband,  (regions A and C for the two distributions respectively). The *rightmost histogram* bin includes overflow. The *small lower plots* show the ratio of normalized sideband to signal yields (Color figure online)

The predicted yield in region D is given by

6 $$ N_{\mathrm{D},\mathrm{pred}} = \frac{ N_{\mathrm{B}} \times N_{\mathrm{C}} }{ N_{\mathrm{A}} }, $$

where *N*_*i*_ is the yield in region *i*, and the predicted yield in region D′ is defined similarly. This procedure is equivalent to using the  distribution of the *z*_ANN_ sideband regions (A, B, and B′) as a template for the  distribution of events with high *z*_ANN_ (C, D and D′), normalized using the yields in regions A and C. We test this estimation procedure using SM simulation: Fig. 12 (top) shows that the  distributions for low and high *z*_ANN_ are similar.

If a signal is present, it enters primarily in the signal regions D and D′, but there are also significant contributions relative to the SM in regions B and B′, somewhat increasing the predicted backgrounds in D and D′. This effect is accounted for in the final results.

Table 8 summarizes the event yields in the sideband subtraction regions for the various components of the SM background. The W+jets and $\mathrm{t}\overline{\mathrm{t}}$ dominate in all the regions, though their relative proportion varies. The W+jets events are most important at low *z*_ANN_ since *M*_T_, which largely drives *z*_ANN_, tends to peak near the W-boson mass. Because the W bosons (and hence their daughters) can be highly boosted, these events extend to very high values of . As seen in Fig. 10, $\mathrm{t}\overline{\mathrm{t}}$ events are more likely to have high values of *z*_ANN_ than are W+jets events; this is because of the presence of dilepton $\mathrm{t}\overline{\mathrm{t}}$ events, in which both W bosons (from the top quark pair) decay leptonically, but only one lepton is identified (dilepton (*ℓ*)), giving large *M*_T_. There is also a small contribution from events in which the lepton comes from the decay of a *τ* produced from a top quark decay, with the other top quark decaying either leptonically (dilepton (*τ*→*ℓ*)) or hadronically (single *τ*). The remaining small backgrounds come from single-top-quark, QCD multijet and Z+jets events.

**Table 8:**
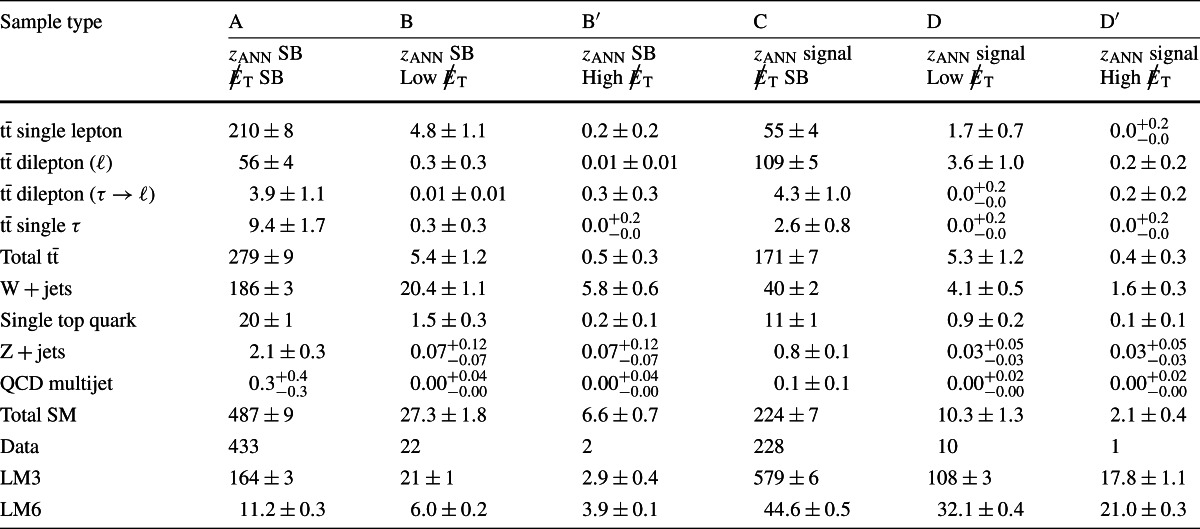
Event yields for the sideband (SB) and signal regions used in the ANN method. The uncertainties listed are statistical only

There are too few events in the simulated QCD multijet and Z+jets samples to populate the high  regions (B, B′, D and D′). For the results quoted in Table 8 for QCD multijet and Z+jets events, we employ an extrapolation technique based on loosening the *z*_ANN_ and  requirements. The extrapolated numbers for all the regions are consistent with those obtained from the simulated samples. The simulated yields in the sideband and signal regions indicate that QCD multijet and Z+jets events are negligible.

**Fig. 13:**
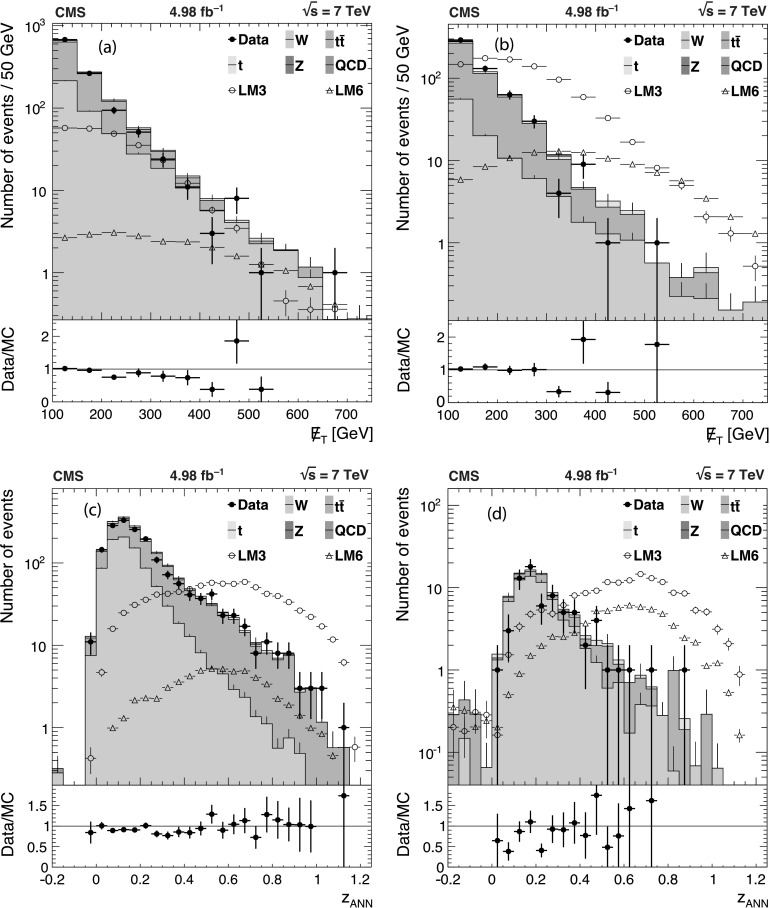
Distributions of  for (**a**) 0.2<*z*_ANN_<0.4 and (**b**) *z*_ANN_>0.4, and distributions of *z*_ANN_ for (**c**)  and (**d**) . The samples shown are data (*solid circles*), simulated SM (*stacked shaded histograms*), LM3 (*open circles*), and LM6 (*open triangles*) events. The *small plot beneath each distribution* shows the ratio of data to simulated SM yields

The total SM simulation yields agree well with data in all regions, suggesting that the data share the main features described above. The *z*_ANN_ and  distributions are shown in Fig. 13.

### Results of the ANN method

Figure 12 (top) shows the results of applying the background estimation method to the SM simulation. We find that the method correctly predicts the background within a factor of $\kappa= \mathrm{D}^{\prime}/ \mathrm{D}^{\prime}_{\mathrm{pred}}$ of 0.82±0.12 (stat.) in the low- signal region and 0.69±0.16 (stat.) in the high- signal region. The modest deviation from unity results from a correlation between *z*_ANN_ and  that arises because the W+jets background, which extends to large  values, dominates in the *z*_ANN_ sideband (because it tends to have *M*_T_ near the W mass), whereas dileptonic $\mathrm{t}\overline{\mathrm{t}}$ events, with their somewhat softer  spectrum, dominate in the *z*_ANN_ signal region.

Figure 12 (bottom) shows the  distributions of the data in the high and low *z*_ANN_ regions, after normalizing in the region  (A and C). Because the SM simulation appears to describe the data well, with, for example, consistent exponential decay constants describing the  distributions in the ANN sidebands, we choose to scale the background prediction of the data by *κ*. The uncertainty in the background from the relative cross sections of SM processes and other effects is quantified in Sect. 8. In the low- signal region, we expect 9.5±2.2 (stat.) events, and in the high- signal region 0.7±0.5 (stat.) events. The observed yields are 10 and 1 events, respectively, consistent with the background prediction.

## Systematic uncertainties

Systematic uncertainties affect both the background estimates and the signal efficiencies. The sources of systematic uncertainty in the background predictions vary among the three methods, both because the final event selections differ and because the background estimation methods themselves differ. The systematic uncertainties stem from lack of perfect knowledge of the detector response and from uncertainties in the properties of the SM backgrounds. Common uncertainties for all methods are described in Sect. 8.1, while details that are specific to each method are given in Sects. 8.2, 8.3, and 8.4 for the LS, *L*_P_, and ANN methods, respectively. Tables 9, 10, and 11 list the main uncertainties associated with each method. The systematic uncertainties affecting the signal efficiency and luminosity, which are largely common to all methods, are described in Sect. 8.5.

**Table 9:** Sources of systematic uncertainties for the LS method and their effects on the background prediction in bins of . The full list of systematic uncertainties is given for *H*_T_>750 GeV, and the total uncertainties are shown for *H*_T_>500 GeV and *H*_T_>1000 GeV. Each uncertainty is expressed as a change in the ratio of the predicted to the true number of events (evaluated with simulation). Uncertainties associated with the dilepton and QCD backgrounds are discussed in the text. The total uncertainty is the individual uncertainties summed in quadrature

[GeV]	[250–350) (%)	[350–450) (%)	[450–550) (%)	≥550 (%)
*H*_T_>750 GeV				
Jet and energy scale	11	13	14	16
Lepton efficiency	1	1	1	1
Lepton *p*_T_ scale	1	2	6	2
$\sigma(\mathrm{t}\overline{\mathrm{t}})$ and *σ*(W)	1	1	4	4
W polarization in $\mathrm{t}\overline{\mathrm{t}}$	1	1	1	1
W polarization in W+jets	3	4	12	11
Z+jets background	4	4	4	4
SM simulation statistics (K-factors)	4	7	12	17
Total systematic uncertainty	13	16	24	27
*H*_T_>500 GeV				
Total systematic uncertainty	16	18	29	30
*H*_T_>1000 GeV				
Total systematic uncertainty	15	18	28	32

**Table 10:** Sources of systematic uncertainties for the *L*_P_ method and their effects on the background prediction in bins of $S_{\mathrm{T}}^{\mathrm{lep}}$ for the muon and electron channels. The full list of systematic uncertainties are given for the range 500<*H*_T_<750 GeV, and the total uncertainties are shown for the two ranges 750<*H*_T_<1000 GeV and *H*_T_>1000 GeV. The total uncertainty is the individual uncertainties summed in quadrature

$S_{\mathrm{T}}^{\mathrm{lep}}$ range [GeV]	[150–250) (%)		[250–350) (%)		[350–450) (%)		≥450 (%)	
Channel	*μ*	e	*μ*	e	*μ*	e	*μ*	e
500<*H*_T_<750 GeV								
Jet and energy scale	6	6	4	5	5	9	9	9
Lepton efficiency	5	5	5	2	3	1	1	2
Lepton *p*_T_ scale	0	–	1	–	1	–	2	–
$\sigma(\mathrm{t}\overline{\mathrm{t}})$ and *σ*(W)	3	1	1	1	1	2	1	1
W polarization in $\mathrm{t}\overline{\mathrm{t}}$	0	1	1	1	1	1	1	2
W polarization in W+jets	2	1	2	1	2	3	3	4
resolution	2	2	1	1	1	2	4	4
$\mathrm{t}\overline{\mathrm{t}}$ (*ℓℓ*)	5	5	5	5	3	3	1	1
SM simulation statistics	1	1	2	2	4	5	6	7
Total systematic uncertainty	11	10	9	8	8	12	13	13
750<*H*_T_<1000 GeV								
Total systematic uncertainty	9	12	10	11	13	13	12	13
*H*_T_>1000 GeV								
Total systematic uncertainty	10	15	13	15	20	18	16	20

**Table 11:** Sources of systematic uncertainties for the ANN method and their effects on the background prediction in bins of . The total uncertainty is the individual uncertainties summed in quadrature

range [GeV]	[350–500) (%)	≥500 (%)
Jet and energy scale	3	4
Lepton *p*_T_ scale	3	5
Lepton efficiency	0.3	0.4
$\sigma(\mathrm{t}\overline{\mathrm{t}})$ and *σ*(W)	3	2
W polarization in W+jets	1	3
W boson *p*_T_ spectrum in W+jets	10	2
$\mathrm{t}\overline{\mathrm{t}}$ (*ℓℓ*)	1	7
Other backgrounds	1	1
SM simulation statistics	15	23
Total systematic uncertainty	19	26

### Common uncertainties in the background predictions

The jet energy scale (JES) and its effect on  in the event can affect the *H*_T_ and  distributions and can also lead to differences between the lepton *p*_T_ spectrum and  spectrum. To understand the effects of energy-scale variations, we vary the jet energy scale as a function of *p*_T_ and *η* by amounts determined in independent studies of jet energy scale uncertainties [38], and corresponding to 2 GeV or less for jets with *p*_T_>40 GeV, and then recompute *H*_T_ and . We also vary the energy scale of “unclustered” calorimeter deposits by 10 % to take into account energy not clustered into jets (this effect is very small).

The uncertainty in the lepton efficiency accounts for differences between data and simulation and uncertainties in the trigger efficiencies. The lepton efficiencies are studied using a sample of lepton pairs with invariant mass close to the Z peak, in which one lepton satisfies tight selection criteria, and the second, reconstructed with relaxed criteria, serves as a probe of the tighter reconstruction and isolation requirements (“tag-and-probe” method [43]). Discrepancies between the data and simulation for electrons are maximal at low *p*_T_ (10 % effect at around 20 GeV), and we reweight events as a function of lepton *p*_T_ to quantify the effect. The total lepton efficiency in data is described by simulation with an accuracy of 3 %. Studies of the trigger that separately determine the efficiencies of the $H_{\mathrm{T}}^{\mathrm{trigger}}$, , and lepton requirements show that the lepton inefficiencies dominate, and amount to 2 % to 3 % for leptons that are reconstructed successfully offline. Muon *p*_T_ scale uncertainties are obtained from the study of the *q*/*p*_T_ (transverse curvature with sign given by the electric charge *q*) distribution of muons in Z events in data. By comparing the *q*/*p*_T_ distribution of positive and negative muons it is possible to quantify the amount of bias in the measurement of *q*/*p*_T_.

The relative amount of $\mathrm{t}\overline{\mathrm{t}}$ and W+jets background affects each analysis method through corrections obtained from simulation. The contributions from $\mathrm{t}\overline{\mathrm{t}}$ and W+jet have not been specifically measured in the narrow region of phase space studied in this analysis and their relative contribution must be evaluated. The $\mathrm{t}\overline{\mathrm{t}}$ cross section is validated using an algorithm based on the reconstructed top-quark masses for both the hadronic and the leptonic top-quark decays. The uncertainty in the $\mathrm{t}\overline{\mathrm{t}}$ cross section is determined by comparing yields in data and simulation after a selection based on top mass variables. The W+jets cross section is validated by comparing event yields between data and simulation in Z+jets events in a dedicated dilepton event selection with similar kinematics. We assign an uncertainty to the W+jets cross section based on the agreement of the data and simulation in the Z+jets sample. Using the uncertainties obtained for the $\mathrm{t}\overline{\mathrm{t}}$ and W+jets cross sections, we probe different relative contributions of $\mathrm{t}\overline{\mathrm{t}}$ and W+jets events in our sample and the effect on our background predictions.

Uncertainties in the polarization fraction for the W boson, either in $\mathrm{t}\overline{\mathrm{t}}$ or W+jets events, must be taken into account. For the W polarization in $\mathrm{t}\overline{\mathrm{t}}$ events, the theoretical uncertainties are very small (see Sect. 3) and have negligible effect on the background predictions. The W polarization in W+jets events, which is described in more detail in Sect. 3, is more complicated than in $\mathrm{t}\overline{\mathrm{t}}$ production. In this case, we consider the effect of conservative variations of the helicity fractions in bins of W-boson *p*_T_ and *η* with respect to the theoretical NLO calculations [36].

For the dilepton $\mathrm{t}\overline{\mathrm{t}}$ background, $\mathrm{t}\overline{\mathrm{t}}$ (*ℓℓ*), the uncertainties are evaluated somewhat differently for the different methods. In the *L*_P_ and ANN methods this background is evaluated together with the same control sample as for the main single-lepton background prediction. Uncertainties in the prediction can arise from finite detector acceptance, inefficient lepton identification, and cross section uncertainties. In the LS method the dilepton $\mathrm{t}\overline{\mathrm{t}}$ background is not predicted using the single-lepton background prediction and separate control samples must be used. Thus the uncertainties for the dilepton $\mathrm{t}\overline{\mathrm{t}}$ background are estimated separately and described in the next section.

The small residual QCD multijet background is probed by inverting the requirement on $I_{\mathrm{rel}}^{\mathrm{comb}}$ or the electron selection criteria to obtain QCD dominated control samples. Contamination from $\mathrm{t}\overline{\mathrm{t}}$ and W+jet events in these control samples must be considered and the uncertainties on their cross sections are the dominant uncertainty for these methods.

The Z+jets contribution to the signal regions is very small and uncertainties on this background prediction come from lepton efficiency and cross section uncertainties. In addition, for the LS method there is a small Z+jets contamination to the single-lepton control sample, which must be subtracted, and lepton efficiency and cross-section uncertainties are considered for this as well.

### Lepton Spectrum method background prediction uncertainty

For the LS method, the systematic uncertainties for each of the different background predictions from control samples in data (1 *ℓ*, dilepton, 1 *τ*, QCD, and Z+jets) are included in Tables 2, 3, and 4. To determine the systematic uncertainties for the largest source of background, 1-*ℓ* events (arising from $\mathrm{t}\overline{\mathrm{t}}$, W+jets, and single-top processes), we evaluate deviations for the -dependent correction factor, which is determined from simulation and applied to the 1-*ℓ* background prediction (see Sect. 5.2). Table 9 gives a breakdown of the contributions of the systematic uncertainties for the 1-*ℓ* prediction in bins of  and for *H*_T_>750 GeV. The uncertainties in the 1-*ℓ* prediction for the *H*_T_>500 GeV and *H*_T_>1 TeV signal regions are similar to those listed in Table 9. The largest source of uncertainty arises from the potential difference in the muon *p*_T_ and the  scales, because the muon *p*_T_ spectrum is used to predict the  spectrum. The statistical uncertainties in the correction factors (denoted as K-factors in Table 9) for the 1-*ℓ* method are slightly smaller than the combined systematic uncertainty of the correction factor. Table 9 does not include an uncertainty from jet resolution effects because this is taken into account by the smearing of the lepton *p*_T_ spectra by QCD multijet  templates (described in Sect. 5.2). For the purposes of setting limits, the total systematic uncertainty in the 1-*ℓ* background prediction is treated as correlated across all bins in .

Tables 2, 3, and 4 also list the non-single-lepton backgrounds, which account for about 25 % of the total, with a relative uncertainty of 5–10 % in the lowest- bin and about 30 % in the highest- bin. For the dilepton prediction of lost and ignored leptons (described in Sect. 5.3) the main sources of systematic uncertainty arise from the lepton reconstruction and identification efficiencies and the top-quark *p*_T_ spectrum. The uncertainties on the lepton efficiencies are described in Sect. 8.1, and the uncertainty associated with the top-quark *p*_T_ spectrum is determined from varying the fraction of events in the tail of this distribution in simulation in a manner consistent with the uncertainty in this tail as observed in data. This uncertainty is then propagated through the background determination procedure.

### Lepton Projection method background prediction uncertainty

For the *L*_P_ method, the estimate of the total number of events expected from SM processes in the signal region, $\mathrm{N}_{\mathrm{SM}}^{\mathrm{pred}}(L_{\mathrm{P}}<0.15)$, relies on the knowledge of the translation factor, *R*_CS_, as well as the number of events observed in the control region, subtracted for the QCD background, N_data_(*L*_P_>0.3). There are, therefore, two sources of uncertainty in this estimate: uncertainties in the number of events from EWK processes in the control region and uncertainty in *R*_CS_. The relative change on the predicted background from each source of systematic uncertainty is listed in Table 10 for both muons and electrons. The largest uncertainty for high $S_{\mathrm{T}}^{\mathrm{lep}}$ bins is the statistical uncertainty in the data in the control region. The second largest uncertainty comes from the JES uncertainty. The effect from the JES uncertainty is larger in the electron channel, since the JES affects also the shape of the *L*_P_ distribution used in the fit of the control region. The uncertainty in the resolution of the measurement of the hadronic energy recoiling against the lepton and  is evaluated conservatively by smearing the total recoil energy in simulation by an additional 7.5 % along the direction of the recoil and by 3.75 % in the direction orthogonal to the recoil. This decreases the resolution more than 10 % for the high recoils (above 250 GeV) of the signal region and thus covers the difference between data and simulation.

### ANN method background prediction uncertainty

For the ANN method, the systematic uncertainty in the background prediction is dominated by the statistics of the simulation, which probes for bias in the background estimation. Another important uncertainty comes from the *p*_T_ spectrum of the W boson in W+jets events, since it affects the  distribution of these events, which preferentially populate the *z*_ANN_ sideband. To assess the impact, we reweight the *p*_T_ spectrum of W boson events, using the differences in the *p*_T_ spectra of Z bosons in data and simulation as a guide. This uncertainty is driven by the statistics of the Z+jets sample. The relative proportions of W+jets and $\mathrm{t}\overline{\mathrm{t}}$ events differ in the *z*_ANN_ signal and sideband regions so the background prediction depends on their relative cross sections. Those $\mathrm{t}\overline{\mathrm{t}}$ events with two leptons in the final state, only one of which is observed, have large  and are the source of most SM events in the signal region. In addition to the $\mathrm{t}\overline{\mathrm{t}}$ cross section, this background depends on lepton acceptance and identification inefficiencies. Additional sources of systematic uncertainty are the hadronic and leptonic energy scales. Table 11 summarizes these uncertainties.

### Signal efficiency and other multiplicative uncertainties

The systematic uncertainty in the signal yield arises from the uncertainty in the signal efficiency. In general, this uncertainty is correlated across  or $S_{\mathrm{T}}^{\mathrm{lep}}$ bins. The JES component of the signal efficiency uncertainty is computed separately for each model point in CMSSM and simplified model parameter space and is correlated with the JES uncertainty in the single-lepton background prediction. The systematic uncertainties in the signal efficiency associated with lepton reconstruction and the trigger amount to 3 %. The uncertainty in the integrated luminosity is 2.2 % [44]. The systematic uncertainty in the signal efficiency, not including the JES component, is 6 % for each of the analyses.

## Results and interpretation

The LS, *L*_P_, and ANN methods each yield SM background predictions that are consistent with the number of events observed in data. We therefore proceed to set exclusion limits on SUSY model parameters. All limits are computed using the modified-frequentist CLs method [45] with a one-sided profile likelihood test statistic. To interpret the absence of an observed signal, three complementary approaches are used.

### Constraints on CMSSM parameter space

First, we scan over models in the CMSSM and determine whether the number of events predicted at each model point in parameter space can be excluded by the measurements. This procedure relies on the fact that the CMSSM parameter space can be described with just five parameters, and we fix three of them to commonly used values (*A*_0_=0 GeV, *μ*>0, tan*β*=10). Each model point has a complete SUSY particle spectrum and a well defined cross section, which typically involves several production subprocesses. The CMSSM simulated samples are initially generated using leading-order cross sections. At each point in CMSSM parameter space, the predicted yields for each production subprocess (e.g., $\mathrm {g}\mathrm{g}\to \widetilde{\mathrm{g}}\widetilde{\mathrm{g}}$) are corrected using the NLO cross sections discussed in Ref. [46]. Using the observed yield in data and the predicted background, we determine whether the CMSSM yield for the particular model point can be excluded at the 95 % confidence level (CL). This procedure is complicated by the fact that the control regions in data could potentially be contaminated by signal events. This effect is taken into account for each model by removing the expected contribution to the predicted background arising from signal contamination of the control regions.

**Fig. 14:**
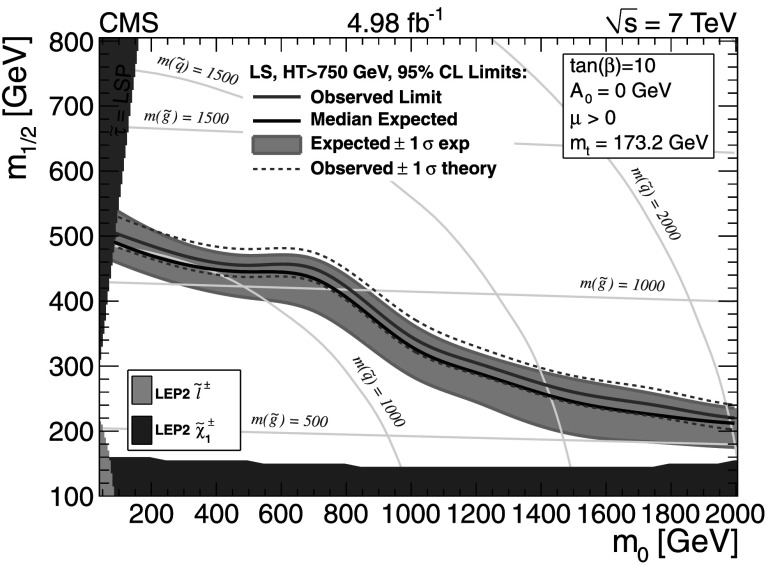
LS method: exclusion region in CMSSM parameter space for the *H*_T_>750 GeV selection

**Fig. 15:**
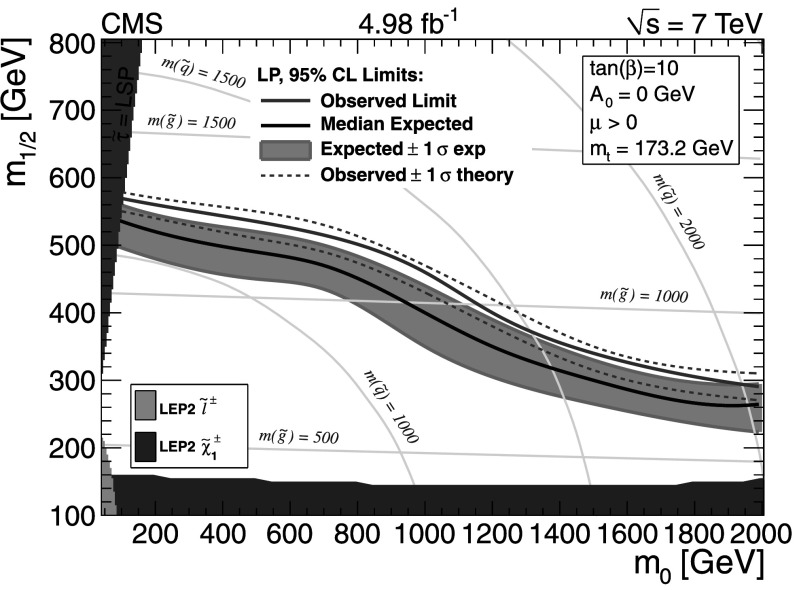
*L*_P_ method: exclusion region in CMSSM parameter space for all *H*_T_ bins combined

**Fig. 16:**
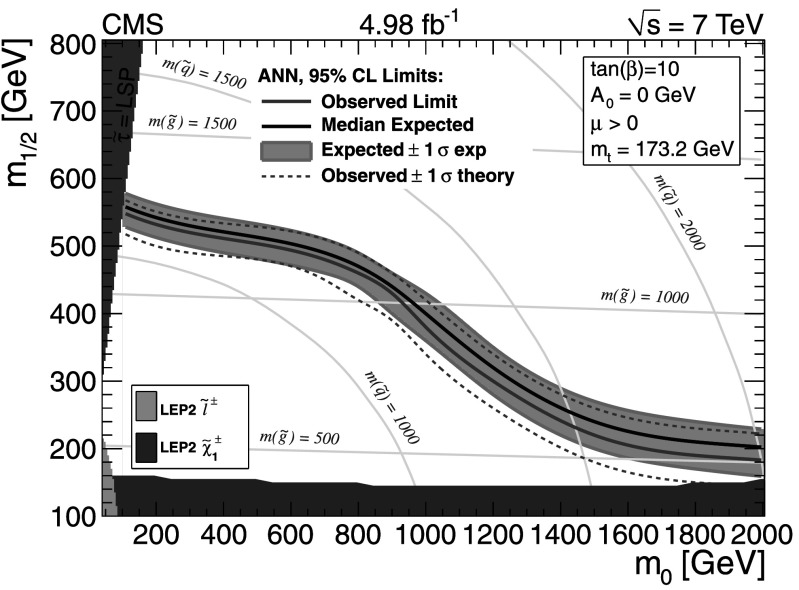
ANN method: exclusion region in CMSSM parameter space

**Fig. 17:**
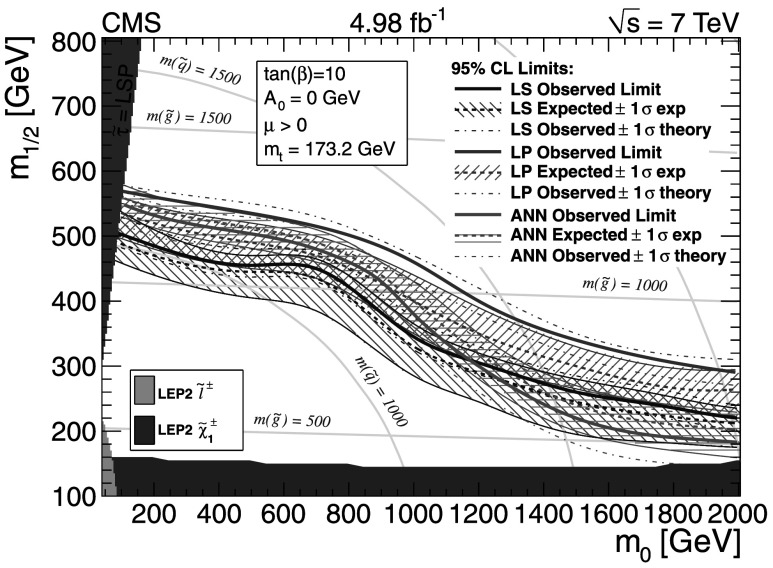
Exclusion region for the LS, *L*_P_, and ANN methods in CMSSM parameter space. Results from the low- and high- signal regions are combined

Figures 14, 15, and 16 show the CMSSM exclusion region [47] for the three background estimation methods, evaluated in the *m*_1/2_ vs. *m*_0_ plane, with the values of the remaining CMSSM parameters fixed at tan*β*=10, *A*_0_=0 GeV, and *μ*>0. Figure 17 displays all of the results together. The excluded regions are below the plotted curves, corresponding to SUSY particle masses below certain values. For reference, the plots display curves of constant gluino and squark masses. The lines of constant gluino mass are approximately horizontal with $m(\widetilde{\mathrm{g}})\approx 2.5~m_{1/2}$. Lines of constant squark mass are strongly curved in the *m*_1/2_ vs. *m*_0_ plane. At low *m*_0_, the analyses exclude gluinos with masses up to about 1.3 TeV, but the sensitivity falls with increasing *m*_0_. To determine the one standard deviation (*σ*) theoretical uncertainty on the observed limit, the signal yields are recomputed after changing each of the process-dependent SUSY production cross sections at each model point by ±1*σ* of their uncertainty arising from the parton distribution functions and renormalization and factorization scales [46].

### Constraints on simplified model parameter space

The second approach to interpretation is based on the use of simplified models [14, 15], which provide a more generic description of new physics signatures. Such models do not include a full SUSY particle spectrum, but instead include only the states needed to describe a particular set of decay chains of interest. Rather than excluding a model, the procedure is to calculate cross section upper limits on a given topological signature. (Such cross section limits can, however, be converted into limits on particle masses within the assumptions of the particular model.) Because simplified models do not describe a full SUSY spectrum, the number of free parameters is small. Furthermore, the parameters are simply the masses of the SUSY particles, in contrast to the grand-unified-theory-scale parameters used in the CMSSM. An advantage of simplified models is that, as a consequence, certain relationships between particle masses that arise with the CMSSM no longer hold, and the spectra can be much more generic.

**Fig. 18:**
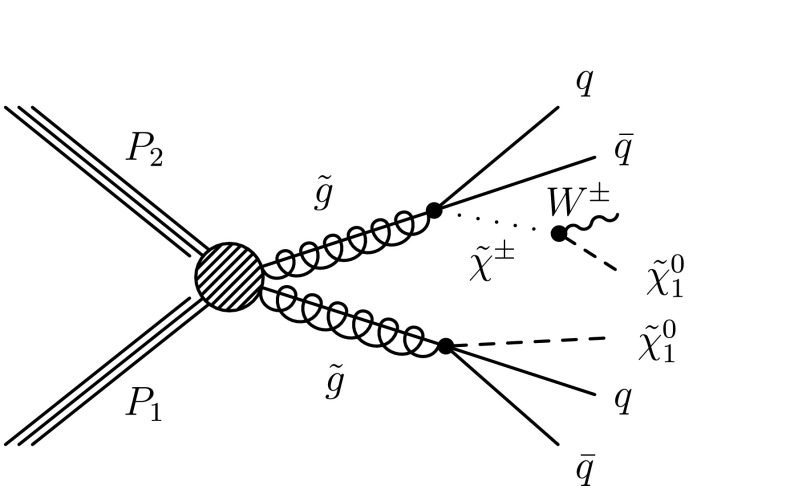
Diagram for production and decay in the T3w simplified model

**Fig. 19:**
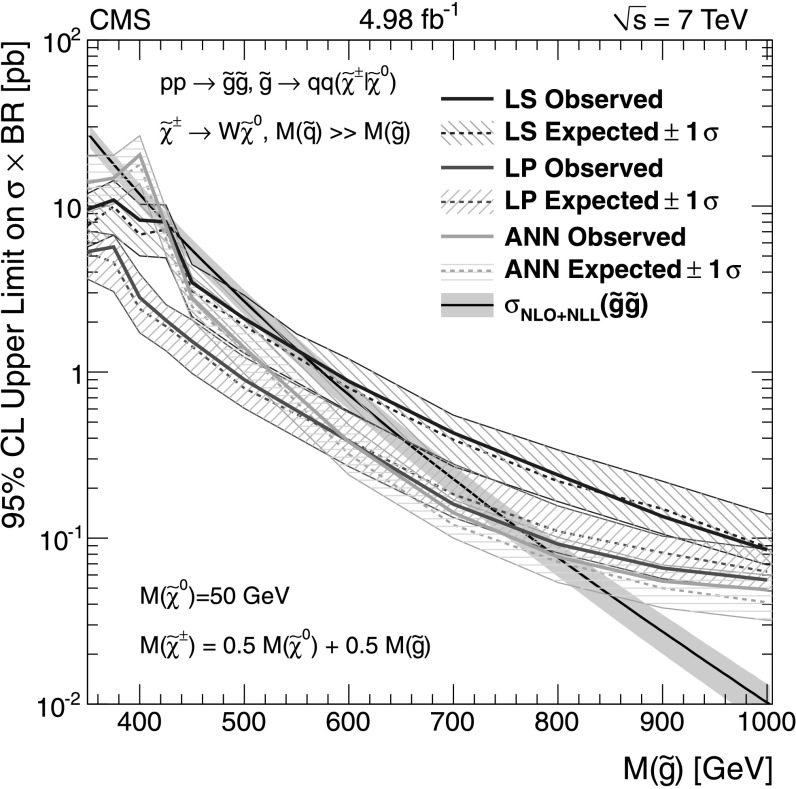
Excluded cross sections for the LS, *L*_P_, and ANN methods for the T3w simplified model

We consider the “Topology 3 weakino” (T3w) simplified model, which involves the production of two gluinos and their decay via the mechanism shown in Fig. 18. One gluino is forced to decay into two quark jets plus the LSP ($\widetilde{\chi}^{0} $) via the three-body decay $\widetilde{\mathrm{g}}\to\mathrm{q}\bar{\mathrm{q}}\widetilde{\chi}^{0}$, while the other gluino decays via $\widetilde{\mathrm{g}}\to\mathrm{q}\bar{\mathrm{q}}^{\prime}\widetilde{\chi }^{\pm}$, followed by $\widetilde{\chi}^{\pm}\to\mathrm{W}^{\pm}\widetilde{\chi}^{0}$. The W^±^ boson can then decay leptonically. The T3w model is specified by masses of the gluino, the LSP ($\widetilde{\chi}^{0}$), and an intermediate chargino ($\widetilde{\chi}^{\pm}$). We calculate cross section limits as a function of $M(\widetilde{\mathrm{g}})$, assuming a fixed value for the LSP mass $M(\widetilde{\chi }^{0})=50~\mathrm{GeV}$ and setting the chargino mass according to $M(\widetilde{\chi}^{\pm})=0.5( M(\widetilde{\chi}^{0}) + M(\widetilde {\mathrm{g}}))$. The nominal production cross section for the gluino pair production mechanism is given in Ref. [46]. Figure 19 shows the cross sections excluded by each method for this model. The limits fluctuate significantly at low $M(\widetilde{\mathrm{g}})$ because of the low signal efficiency in this region.

### Alternate model exclusions

The data can be interpreted using a third approach, which is applicable to models that do not fall within the scope of either the CMSSM or the simplified model discussed in this section. A model builder can investigate the sensitivity of any one of the three methods presented in this paper to a given signal hypothesis by applying the event selection requirements listed in Table 1, together with the final requirements that define the signal regions. We provide a simple efficiency model for the most important observables used in the event selections. The efficiency model can then be applied to a basic (pythia) simulation of the signal process.

The efficiency model is based on parametrizations of the efficiencies for the event selection requirements with respect to the main reconstruction objects and quantities, such as *H*_T_, , and lepton *p*_T_. The efficiency of the analysis for a given model can be estimated by applying these individual reconstruction efficiencies, which are given as a function of the most important parameter (such as lepton *p*_T_), to the corresponding kinematic distributions in the model. This procedure would then yield an estimate for the number of signal events from the model. Finally, the sensitivity of the analysis to the model can be obtained by comparing the yield of signal events obtained in this manner with the background yields given in this paper. Kinematic correlations (which can be model dependent) are not taken into account, but this approach nonetheless provides a first approximation to the sensitivity.

The efficiencies for each analysis object are described using “turn-on” curves, which are simply error functions,

7 $$ \epsilon(x) = \epsilon_\mathrm{plateau} \frac{1}{2} \biggl[ \operatorname{erf} \biggl(\frac{x-x_\mathrm{thresh}}{\sigma}\biggr) + 1 \biggr], $$

where *x* represents the variable most relevant for the reconstruction of the particular object. The error function is parametrized in terms of the plateau efficiency, *ϵ*_plateau_; the turn-on threshold, *x*_thresh_; and the characteristic width of the turn-on region, *σ*. These parameters are obtained by fitting simulated event samples as a function of the true (generated) value.

The selection efficiency associated with the lepton reconstruction, identification, and isolation requirements is estimated as a function of lepton *p*_T_ by considering muons and electrons (including those from *τ* decay) generated in the pythia-simulated hard-scattering process. The lepton isolation requirement has a large effect on the efficiency, which consequently depends on the number of jets in the event. To reduce the model dependence arising from this effect, two categories of leptons are considered. First, we assign zero efficiency to leptons that are within Δ*R*<0.4 of a quark or gluon with *p*_T_>40 GeV in the hard-scattering process. The efficiency for the remaining leptons is described by a turn-on curve whose parameters are listed in Table 12. The efficiencies are specified for both the lepton selection and for the lepton veto.

**Table 12:** Efficiency-model parameters for lepton efficiencies as a function of *x*≡*p*_T_. The leptons are required to lie within the fiducial region and must satisfy the *p*_T_ thresholds specified in Table 1

Lepton	*ϵ*_plateau_	*x*_thresh_ [GeV]	*σ* [GeV]
Muon (signal)	0.86	2.7	65
Muon (veto)	0.90	−17	75
Electron (signal)	0.74	20	61
Electron (veto)	0.83	2.3	54

The number of jets and the resulting *H*_T_ value for each event are computed using information available at the generator level. The same clustering algorithm used to reconstruct jets in the data is applied to the generator-level particles. The resulting generator-level jets are required to satisfy Δ*R*>0.3 with respect to the leptons described above. The  variable is estimated at the generator level from the transverse momenta of neutrinos and any new weakly interacting particles, such as the $\widetilde{\chi}^{0}$. The parametrizations of the efficiency turn-on curves for the *H*_T_ and  requirements are listed in Tables 13 and 14, respectively. For the requirements used with the LS method, the information given in these tables generally reproduces the efficiency from full simulation to within about 15 %.

**Table 13:** Efficiency-model parameters for *x*≡*H*_T_

Threshold	*ϵ*_plateau_	*x*_thresh_ [GeV]	*σ* [GeV]
*H*_T_≥400 GeV	1.00	396	65
*H*_T_≥500 GeV	1.00	502	66
*H*_T_≥750 GeV	1.00	760	68
*H*_T_≥1000 GeV	1.00	1013	80

**Table 14:** Efficiency-model parameters for

Threshold	*ϵ*_plateau_	*x*_thresh_ [GeV]	*σ* [GeV]
	1.00	103	41
	0.99	266	41
	0.98	375	45
	0.97	485	48
	0.94	537	44
	0.96	597	59

In the *L*_P_ method, the variables *L*_P_ and $S_{\mathrm{T}}^{\mathrm{lep}}$ are functions of lepton *p*_T_ and . The modeling of lepton *p*_T_ is described above. To emulate , one needs to apply both a scale shift and smearing to the generated  value. The  scale factor is . The value of  is about 0.2 at . It falls linearly to about 0.06 at , and it remains at 0.06 for .

In the ANN method, the preselection requirements on *H*_T_ and  are 400 and 100 GeV, respectively. The signal regions are specified by  and  together with *z*_ANN_>0.4, where *z*_ANN_ is a function^1^ of *n*_jets_, *H*_T_, Δ*ϕ*(j_1_,j_2_), and *M*_T_. The efficiency turn-on curve for *z*_ANN_>0.4 is approximated by the parameter values *ϵ*_plateau_=0.98, *x*_thresh_=0.41, and *σ*=0.1.

With these additional procedures, the emulation of the efficiencies for the *L*_P_ and ANN methods is found to be accurate to within ∼15 %, as for the LS method.

## Summary

Using a sample of proton–proton collisions at $\sqrt{s}=7$ TeV corresponding to an integrated luminosity of 4.98 fb^−1^, we have performed a search for an excess of events with a single, isolated high-*p*_T_ lepton, at least three jets, and large missing transverse momentum. To provide a robust and redundant determination of the SM backgrounds, three methods are used, each of which relies primarily on control samples in the data.

The Lepton Spectrum (LS) method exploits the relationship between two key observables, the lepton *p*_T_ distribution and the  distribution. In the dominant SM background processes, which have a single, isolated lepton, this connection arises from the fact that the lepton and neutrino are produced together in the two-body decay of the W boson, regardless of whether the W is produced in $\mathrm{t}\overline{\mathrm{t}}$ or W+jets events. In many SUSY models, however, the  is associated with the production of two neutralinos, which decouples  from the lepton *p*_T_ spectrum. Smaller backgrounds arising from $\mathrm{t}\overline{\mathrm{t}}$ dilepton events, from *τ*→*ℓ* decays in $\mathrm{t}\overline{\mathrm{t}}$ or W+jets events, and from QCD multijet processes are also estimated using control samples in the data. In the sample investigated with this method, at least four jets are required, which helps to suppress the background from W+jets events. Nine signal regions are considered, specified by three thresholds on *H*_T_ and three bins of . The observed yields in each region are consistent with the background estimates based on control samples in the data.

The Lepton Projection (*L*_P_) method exploits information on the W-boson polarization in $\mathrm{t}\overline{\mathrm{t}}$ and W+jets events. The dimensionless *L*_P_ variable itself is sensitive to the helicity angle of the lepton from W decay, but it also provides discrimination between signal and background through the ratio of the lepton *p*_T_ and the  values, which is small in SUSY-like events. The $S_{\mathrm{T}}^{\mathrm{lep}}$ variable maps out a diagonal line in the plane of lepton *p*_T_ vs.  and reflects the W transverse momentum for the boosted W boson. The *L*_P_ distributions are studied in bins of $S_{\mathrm{T}}^{\mathrm{lep}}$, and *H*_T_, and at least three jets are required. In each signal region, the data are in agreement with expectations from the SM.

The artificial neural network (ANN) method provides a means to obtain the  distribution of background events in data by constructing a neural network variable *z*_ANN_, which has a very small correlation with . This variable also provides strong discrimination between signal and background events, so that the background regions do not suffer from large signal contamination in the models considered. A key element of the *z*_ANN_ variable is the transverse mass of the lepton- system, but additional variables, such as the number of observed jets, play a role as well. In the ANN analysis, no excess of events is observed in the signal regions with respect to the SM background prediction.

Because these methods probe extreme kinematic regions of the background phase space, the use of redundant approaches provides confidence in the results. Although the LS and *L*_P_ methods both make use of information on the *W*-boson polarization in the background, they are based on different kinematic variables and have different signal regions. The LS method breaks the background into several pieces (single lepton, *τ*→*ℓ*, dilepton, and QCD) and provides a direct background prediction for the  distribution. In contrast, the *L*_P_ method defines a powerful kinematic variable that is used to obtain a global background prediction by extrapolating an overall background shape from a control region into the signal region. The ANN method similarly uses a global approach to estimating the background. The neural-net variable incorporates information used in neither of the other two methods.

The results from each method are interpreted in the context of both the CMSSM and a so-called simplified model, T3w, which has a minimal SUSY particle spectrum. The CMSSM limits exclude gluino masses up to approximately 1.3 TeV in the part of the parameter space in which *m*_0_<800 GeV, but the bound gradually weakens for larger values of *m*_0_. For the T3w simplified model, we obtain cross section upper limits as a function of gluino mass. Finally, we provide an approximate model of our signal efficiency that can be used in conjunction with a simple pythia simulation to determine whether other models can be probed by these data.

## Electronic Supplementary Material

Below are the links to the electronic supplementary material.

annApplication.C (2 kB)

annWeightFile.C (12 kB)

README.txt (3 kB)

## Footnotes

1 See Online Supplementary Material for a C++ function that evaluates the artificial neural network based on the values of the four input variables.
